# Inheritance of resistance to maize lethal necrosis in tropical maize inbred lines

**DOI:** 10.3389/fpls.2024.1506139

**Published:** 2025-01-09

**Authors:** Hilda M. Kavai, Dan Makumbi, Felister M. Nzuve, Vincent W. Woyengo, L. M. Suresh, William M. Muiru, Manje Gowda, Boddupalli M. Prasanna

**Affiliations:** ^1^ International Maize and Wheat Improvement Center (CIMMYT), Nairobi, Kenya; ^2^ Department of Plant Science and Crop Protection, University of Nairobi, Nairobi, Kenya; ^3^ Kenya Agricultural and Livestock Research Organization, Non-Ruminant Research Institute, Kakamega, Kenya

**Keywords:** diallel, combining ability, disease resistance, heritability, maize, maternal, reciprocal, maize chlorotic mottle virus

## Abstract

Maize (*Zea mays* L.) production in sub-Saharan Africa can be improved by using hybrids with genetic resistance to maize lethal necrosis (MLN). This study aimed to assess the general (GCA) and specific combining ability (SCA), reciprocal effects, and quantitative genetic basis of MLN resistance and agronomic traits in tropical maize inbred lines. A total of 182 hybrids from a 14-parent diallel, along with their parents, were evaluated under artificial MLN inoculation and rainfed conditions for 3 years in Kenya. Disease ratings at four time points, grain yield (GY), and other agronomic traits were analyzed using Griffing’s Method 3 and Hayman’s diallel models. Significant (*P* < 0.001) GCA and SCA mean squares were observed for all traits under disease conditions and most traits under rainfed conditions, highlighting the importance of both additive and non-additive genetic effects. However, additive gene action predominated for all traits. Narrow-sense heritability estimates for MLN resistance (*h* ^2^ = 0.52–0.56) indicated a strong additive genetic component. Reciprocal effects were not significant for MLN resistance, suggesting minimal maternal or cytoplasmic inheritance. Four inbred lines showed significant negative GCA effects for MLN resistance and positive GCA effects for GY under artificial MLN inoculation. Inbred lines CKL181281 and CKL182037 (GCA effects for MLN4 = -0.45 and -0.24, respectively) contained the most recessive alleles for MLN resistance. The minimum number of groups of genes involved in MLN resistance was estimated to be three. Breeding strategies that emphasize GCA could effectively be used to improve MLN resistance in this germplasm.

## 1 Introduction

Maize (*Zea mays* L.) is a major cereal crop in sub-Saharan Africa (SSA), where it covers more than 40 million ha of arable land. It is the most important crop for food security, income, and livelihoods for several million smallholder farmers across SSA, especially in eastern and southern Africa where nearly 85% of the maize produced is used as food (Shiferaw et al., 2011). Maize production in SSA was approximately 70 million metric tons in 2020 (FAOSTAT, 2021) and is largely produced by smallholder farmers. Despite its wide cultivation, the average maize yield in SSA is approximately 2.0 t ha^-1^, which is far below the global average of approximately 5.8 t ha^-1^ (Erenstein et al., 2022). The low maize yield is attributed to several factors including the frequent occurrence of drought, poor soil fertility, inadequate use of inputs such as improved seed and fertilizers, the impact of pests and diseases (Prasanna et al., 2021), and parasitic weeds (Menkir et al., 2012). Maize diseases of major economic importance in SSA include fungal (Asea et al., 2002; Menkir and Ayodele, 2005; Vivek et al., 2010; Sserumaga et al., 2020) and viral diseases (van Rensburg et al., 1991; Kyetere et al., 1999). Many of the pests and diseases of maize in SSA have become endemic to the region but there have been cases of new transboundary pests and diseases in recent years, affecting the food security and livelihoods of several million resource-constrained smallholder farmers (Prasanna et al., 2020). An example of a transboundary disease occurrence in Africa was the emergence of maize lethal necrosis (MLN) disease in SSA (Wangai et al., 2012; Mahuku et al., 2015a), a disease that was first reported in the Americas in the 1970s (Niblett and Claflin, 1978; Uyemoto et al., 1980).

Maize lethal necrosis was first reported in Kenya in 2011 but has since spread to several other eastern African countries between 2012 and 2018 (Wangai et al., 2012; Mahuku et al., 2015b; Adams et al., 2014; Lukanda et al., 2014; Mudde et al., 2018). This viral disease is caused by the coinfection of maize plants by the *maize chlorotic mottle virus* (MCMV) and any one of the viruses from the family Potyviridae, such as *sugarcane mosaic virus* (SCMV), *maize dwarf mosaic virus* (MDMV), or *wheat streak mosaic virus* (WSMV) (Redinbaugh and Stewart, 2018; Braidwood et al., 2018; Mwatuni et al., 2020). Recent studies revealed that *Johnson grass mosaic virus* (Stewart et al., 2017) and *maize yellow dwarf virus* (Wamaitha et al., 2018) in association with MCMV, cause MLN. The emergence of MLN in eastern Africa is attributed to the entry of MCMV into the region (Prasanna et al., 2020) since the presence of SCMV was reported in maize in East Africa much earlier (Kulkarni, 1973; Louie, 1980).

MLN has seriously affected maize grain yield and production in eastern Africa to varying levels. Based on community surveys in 2013, total maize loss in Kenya due to the MLN outbreak was estimated at 0.5 million metric tons year^-1^ or 22% of the average annual production, with a value of approximately USD 180 million (De Groote et al., 2016). In 2018, the total quantity of maize lost in Kenya was estimated to be approximately 0.17 million metric tons equivalent to approximately USD 51 million (De Groote et al., 2021). Strategies such as crop rotation to break the disease cycle, the use of clean seed, and vector control have been proposed to manage MLN in SSA. The most economically viable and environmentally sustainable approach to control and manage MLN is the development of resistant or tolerant maize varieties. The economic value of adopting MLN tolerant hybrids was estimated at USD 195–678 million in Kenya and USD 245–756 million in Ethiopia depending on adoption levels of 25–75% (Marenya et al., 2018), suggesting a considerable benefit to farmers in utilizing MLN resistant varieties.

The International Maize and Wheat Improvement Center (CIMMYT) in collaboration with national partners, initiated screening of its germplasm stock and from other sources for resistance to MLN in 2012. A few sources of MLN resistance were identified, and introgression of resistance into CIMMYT’s elite germplasm was initiated. The key lines that have been used for introgression of MLN resistance alleles into CIMMYT’s mid-altitude adapted maize germplasm are the yellow lines KS23-5 and KS23-6 from Kasetsart University, Thailand (Jones et al., 2018; Prasanna et al., 2020). These two lines were extracted from KS23(S)C5, a population that had undergone five cycles of S1 recurrent selection (Jampatong et al., 2010) and have resistance to maize mosaic virus (Brewbaker, 2009). Through pedigree breeding and the use of doubled haploid (DH) technology, several inbred lines have been developed, screened under MLN disease pressure to identify resistant lines with adaptive traits, and utilized for hybrid development. Some of the elite CIMMYT lines have also been converted into MLN resistant versions using the major quantitative trait locus (QTL) (qMLN06.157) from KS23-6 (Prasanna et al., 2020; Murithi et al., 2021).

Knowledge of the genetic basis of resistance to diseases is important in developing breeding strategies. Diallel studies have been used to investigate the genetics of virus disease resistance in maize (Josephson and Naidu, 1971; Loesch and Zuber, 1972; Naidu and Josephson, 1976; Rosenkranz and Scott, 1987; Mutengwa et al., 2012; Beyene et al., 2017; Nyaga et al., 2020). Beyene et al. (2017) and Nyaga et al. (2020) reported that additive gene action is more important than nonadditive gene action for MLN resistance and identified some MLN tolerant inbred lines. Studies on the genetics of maize virus diseases have focused mainly on combining ability for disease parameters but not reciprocal effects that could impact hybrid development plans and disease resistance improvement strategies. To date, studies on MLN in tropical maize have used early to intermediate maturity germplasm. The upper mid-altitude ecologies of Eastern Africa are a major maize production area, where late-maturity maize is the preferred type. However, MLN disease is increasingly affecting this region. Therefore, incorporating resistance to MLN into late-maturity maize germplasm is crucial for sustaining production in this region. Using two different sources of resistance to MLN, alleles for resistance have been introgressed into CIMMYT’s late-maturity maize germplasm suitable for the upper mid-altitude ecology of Eastern Africa. Limited to no information on the combining ability and quantitative genetic parameters of MLN resistance in adapted late maturing tropical maize germplasm has been reported. The objectives of this study were to (i) estimate the combining ability of MLN resistance and other traits among 14 late-maturity inbred lines and assess the importance of reciprocal effects, and (ii) investigate the quantitative genetic basis of MLN resistance in tropical maize.

## 2 Materials and methods

### 2.1 Genetic material

Fourteen inbred lines with varying response to MLN and other characteristics based on field evaluations were selected for this study (**Table 1**). The selected genotypes included five lines conventionally developed through pedigree breeding from biparental crosses involving a known source of resistance to MLN (entries 1−5), six DH lines (entries 6−11), and three drought tolerant inbred lines (entries 12−14). The 14 inbred lines were crossed in a full diallel mating design with reciprocals to generate 182 F_1_ hybrids. The crossing was performed at the Kenya Agricultural and Livestock Research Organization (KALRO) Kiboko Research Center (2°15’S, 37°75’E, 975 m asl), Kenya, in 2019. In the same year, seed of the 14 parental lines and one MLN resistant line (KS23-6) was increased to compose the line trials.

**Table 1:** List of 14 inbred lines used to develop F_1_ hybrids and their reciprocal crosses in a full diallel.

No.	Name	Pedigree	Origin	Growing degree-days (GDD)	Characteristics
1	CKL18912	(CKL05003/KS23-6)-B-9-4-5-2-2-B-B-B	Kenya	970	Tolerant MLN
2	CKL181281	(((KU1403 x 1368)-7-2-1-1-B-B/CML444)-B-8-7-3-2-6-1-2-B-B-B/KS23-6)-B-1-1-4-1-2-B-B-B	Kenya	812	Resistant to MLN
3	CKL181379	(((KU1403 x 1368)-7-2-1-1-B-B/CML444)-B-8-7-3-2-6-1-2-B-B-B/KS23-6)-B-22-3-1-2-3-B-B-B	Kenya	829	Resistant to MLN
4	CKL181847	((CKL05003/La Posta Seq C7-F64-2-6-2-2-B-B-B)DH110-B-B/KS23-6)-B-17-4-1-1-1-B-B-B	Kenya	874	Tolerant to MLN
5	CKL182037	(([CML444/CML395//DTPWC8F31-1-1-2-2-BB]-4-2-2-1-1-B*4/(9071xBabamgoyo)-3-1-BBB)-B-1-2-3-1-3-B/KS23-6)-B-2-1-3-2-1-B-B-B	Kenya	851	Tolerant to MLN
6	CKL176616	((([LZ956441/LZ966205]-B-3-4-4-B-5-B*7/LaPostaSeqC7-F71-1-2-1-1-BBB)-1-7-1-1-BB-B/KS23-5)-B)DH15-B-B-B-B-B	Kenya	926	Tolerant to MLN
7	CKL175951	(CML495/CML341)DH23-B-B-B-B-B	Mexico	903	Late, lowland, drought tolerant (DT)
8	CKL175755	(CML341/CML247)DH84-B-B-B-B-B	Mexico	926	Late, lowland, DT
9	CKL175798	(CML343/CML495)DH57-B-B-B-B-B	Mexico	829	Late, lowland, DT
10	CKL176082	(CML495/CML341)DH3-B-B-B-B-B	Mexico	887	Late, lowland
11	CKDHL120918	(CML445/ZM621B//P100C6-200-1-1-B-B-B-B)@3020-B-B-B-B	Kenya	846	Tolerant to MLN
12	CML585	[KILIMA(ST94)-S5:115/[M37W/ZM607#BF37SR … ]]-B-B-3-5-B-B*4	Kenya	903	Susceptible to MLN; DT
13	CKL14546	(CKL05017/LaPostaSeqC7-F78-2-1-1-1-B-B-B)-B-2-1-2-1-1-B-B-B-B	Kenya	893	Susceptible to MLN; DT
14	CML444	P43-C9-1-1-1-1-1-B-B-B	Zimbabwe	904	Susceptible to MLN; DT

### 2.2 Test locations, experimental design, and trial management

The 182 F_1_ hybrids plus four commercial check hybrids were grown in seven trials that were planted at two locations in Kenya in 2020, 2021, and 2022. The hybrid trial was laid out as a 3 × 62 alpha-lattice (Patterson and Williams, 1976) with two replications. A line evaluation trial was also formed, consisting of the 14 parental lines of the diallel hybrids and one inbred line check. The inbred line trial was laid out as a 3 × 5 alpha-lattice with two replications. In both trials, each experimental unit consisted of one row 5 m long, spaced 0.75 m apart and 0.25 m between plants, resulting in a population density of approximately 53,333 plants ha^-1^. The hybrid and parental line trials were evaluated at the KALRO-CIMMYT MLN screening facility at Naivasha (0°43’S, 36°26’E, 2086 m asl) under artificial inoculation with MLN. There were eight trials (four each of hybrids and lines) planted at Naivasha. The same set of germplasm was evaluated at the KALRO Kakamega Non-Ruminant Research Center (0°16’N, 34°49’E, 1585 m asl) under rainfed and natural foliar disease pressure conditions in six trials (three each for hybrids and lines). The inbred line trials were planted side by side with the hybrid trial at both locations. Standard agronomic and cultural practices were performed as recommended for each location.

### 2.3 Artificial inoculation with MLN causing viruses (MCMV and SCMV) and disease rating

The pure mother cultures of MCMV and SCMV were maintained on susceptible host maize hybrids H614 and PHB30G19, respectively, in separate insect-proof net houses at the KALRO-CIMMYT MLN screening facility. The inoculum was prepared following the protocol described in detail in previous studies (Gowda et al., 2018; Sitonik et al., 2019). Briefly, SCMV and MCMV inocula were initially prepared separately. Then, at the time of inoculation, the two viruses were mixed at a ratio of 4:1 of SCMV and MCMV, respectively. The hybrid and inbred line trials were inoculated with the mixture of SCMV and MCMV twice: first at the 4–5 leaf stage, and a second inoculation was carried out seven days after the first inoculation. A motorized backpack mist blower (Solo 423 Mist Blower, 12 L capacity) was used to deliver the inoculum at a pressure of 10 kg cm^-2^.

Disease rating for response to MLN infection was visually done by observing disease symptoms on all plants in a plot at four time points: at 21 (MLN1), 28 (MLN2), 35 (MLN3), and 42 (MLN4) days after the first inoculation for both inbred lines and hybrids. A scale of 1–9 was used for disease rating, where 1 = completely clean plants with no visible MLN disease symptoms, 3 = mild chlorotic streaks on emerging leaves, 5 = chlorotic streaks and mottling throughout the plant, 7 = severe chlorotic mottling, mosaic, and leaf necrosis throughout the plant, and 9 = complete plant necrosis, and dead plants (Prasanna, 2021; https://hdl.handle.net/10883/21703). The four MLN disease ratings were used to calculate the area under disease progress curve (AUDPC) which is a quantitative measure of disease intensity with time as follows:


$$\text{AUDPC}=\;\sum_{i=1}^{n}\left[\frac{Y_{i}+Y_{i+1}}{2}\right]\left(T_{i+1}+T_{i}\right)$$

where *i* = time of MLN disease rating, *T_i_* is the number of days after inoculation, and Y*_i_* is the MLN disease rating (Shaner and Finney, 1977).

### 2.4 Agronomic and foliar disease data

Days to anthesis (DTA, recorded as days from planting to when 50% of the plants started to shed pollen), days to silking (DTS, recorded as days from planting to when 50% of the plants had emerged silks) and ears per plant (EPP) were recorded. The number of ears per plant (EPP) was obtained by dividing the total number of ears per plot by the number of plants harvested. The response to Turcicum leaf blight (TLB) a major foliar disease in SSA caused by *Exserohilum turcicum* (Pass.) Leonard & Suggs in SSA was recorded under heavy natural disease pressure at KALRO-Kakamega on a scale of 1–9, where 1 = highly resistant, no disease symptoms, and 9 = highly susceptible, with severely necrotic leaves. Kakamega is a high natural disease pressure location used for assessing the response to major maize foliar diseases (Vivek et al., 2010). The foliar disease response was recorded when the crop was at the dough stage. All ears in a single-row plot were harvested, weighed, and representative samples of ears were shelled to determine the percent moisture using a Dickey-John multigrain moisture tester (DICKEY-John Corporation, IL, USA). The grain yield, expressed as t ha^-1^ was calculated from cob weight assuming a shelling percentage of 80% and adjusted to 12.5% moisture content.

### 2.5 Statistical analyses

#### 2.5.1 Analysis of variance

The data were first assessed for homogeneity of variance using Levene’s test before ANOVA, and variances were found to be homogeneous. Analyses of variance were performed using META-R (Alvarado et al., 2020), first by location and then across each separate management condition (artificial MLN inoculation and rainfed conditions). Each location-year combination was considered an environment. Genotypes and locations were considered fixed and random effects, respectively. The linear model used for combined analysis across environments was as follows:


$$Y_{ijrk}=\;\text{μ}\;+\;{\text{α}}_{i}+\;{\text{β}}_{j}+\;{\text{ρ}}_{r}\left({\text{β}}_{j}\right)\;+\;{\text{λ}}_{k}\left[{\text{ρ}}_{r}\left({\text{β}}_{j}\right)\right]\;+\;\text{α}{\text{β}}_{ij}+\;{\text{ϵ}}_{ijrk}$$

where *Y_ijrk_* is the mean of the *i*th genotype, in the *r*th replicate within the *k*th sub-block of the *j*th environment; μ is the grand mean; α*_i_* is the effect of the *i*th genotype; β*_j_* is the effect of the *j*th environment; ρ*_r_* is the effect of the *r*th replicate; ρ*_r_* (β*_j_*) is the effect of the replicates within environments; λ*_k_* is the effect of the *k*th incomplete block; λ*_k_* [ρ*_r_* (β*_j_*)] is the effect of the incomplete blocks within replicates and environments; αβ*_ij_* is the effect of the genotype × environment interaction; and ϵ*_ijrk_* is the residual error. To estimate variance components, all factors were considered random effects. The best linear unbiased predictions (BLUPs) and best linear unbiased estimates (BLUEs) for the genotypes were computed. The broad-sense heritability of recorded traits and disease parameters across environments was estimated according to Hallauer et al. (2010) as follows:


$$H^{2}=\frac{\sigma_{G}^{2}}{\sigma_{G}^{2}+\frac{\sigma_{GE}^{2}}{e}+\frac{\sigma_{e}^{2}}{er}}$$

in which $\sigma_{G}^{2}$ , $\sigma_{G\times E}^{2}$ , and $\sigma_{e}^{2}$ are the genotype, genotype × environment, and residual variance components, respectively, *E* is the number of environments, and *r* is the number of replications.

#### 2.5.2 Diallel analysis

Data from the hybrid trial, excluding that of the commercial hybrid checks, were subjected to diallel analysis following Griffing’s Method 3 Model 1 (Griffing, 1956). The use of Method 3 of Griffing allowed us to investigate the possible influence of reciprocal effects due to cytoplasmic differences and/or cytoplasmic-genic relationships on MLN parameters and other traits. The hybrid source of variation was partitioned into general (GCA) and specific combining ability (SCA), and reciprocal effects. The reciprocal effects were further partitioned into maternal and nonmaternal effects. Diallel analysis was carried out using the AGD-R software for R v3.0 (Rodríguez et al., 2020). The following linear model was used for the analysis:


$$X_{ijkt}=\;\text{μ}\;+t_{i}+b_{ki}+v_{ij}+\;{\left(tv\right)}_{ijt}+e_{ijkt}$$

where X*_ijkt_* = observed trait value (*i* and *j*, are parents; *k*, replication; *t*, environment), μ = population mean; *t_i_* = environment effect; *b_ki_* = block or replication within environment effect; *v_ij_* = genotype effect = *g_i_* + *g_j_* + *s_ij_* + r*_ij_* [where *g_i_* = GCA effect of the *i*th parent, *g_j_* = GCA effect of the *j*th parent, *s_ij_* = SCA effect of the *ij*th F_1_ hybrid, r*_ij =_* reciprocal effect of the *ij*th or *ji*th F_1_ hybrid = *m_i_ + m_j +_ n_ij_* (where *m_i_* = maternal effect of parental line *i*, *m_j_* = maternal effect of parental line *j*, and *n_ij_ =* nonmaternal effect of the *ij*th or *ji*th F_1_ hybrid], (*tv*)*_ijt_* = interaction between genotypes and environments, *e_ijkt_* = residual effect. The relative importance of GCA and SCA was assessed using the ratio of the GCA and SCA sums of squares.

The F_1_ hybrid and parental inbred line data (excluding that of the inbred line check) for MLN disease resistance parameters were further subjected to Hayman’s model (Hayman, 1954a, b) of diallel analysis. Hayman’s diallel analysis provides genetic information on additive and dominance effects of genes, average degree of dominance, distribution of genes, and number of groups of genes which control a trait among others. Hayman’s diallel analysis involves graphical and statistical analyses of array variances and covariances and estimation of genetic parameters (Hayman, 1954a, b; Mather and Jinks, 1971). Briefly, Hayman’s diallel analysis requires the calculation of the variances from all crosses of each parental array (*V_r_*), and the covariance between parents and their crosses in each array (*W_r_*) (Hayman, 1954a). The variances and covariances were calculated and used to construct a *W_r_-V_r_* graph. In addition, quantitative genetic parameters for MLN resistance were estimated. Hayman’s diallel analysis was carried out using the SASHAYDIALL program (Makumbi et al., 2018a) in SAS (SAS Institute, 2016).

## 3 Results

### 3.1 ANOVA under artificial MLN conditions

The combined ANOVA across four seasons under artificial MLN inoculation showed significant (*P* < 0.001) environment (E) and genotype (G) mean squares for all agronomic traits and disease parameters (**Table 2**). The G × E interaction was significant for all traits except EPP. Both GCA and SCA mean squares were significant (*P* < 0.001) for all the traits measured under artificial MLN inoculation. The differences between the F_1_ hybrids and their reciprocals were significant for DTA, GY, and AUDPC. Partitioning of the reciprocal source of variation into maternal and nonmaternal effects revealed that maternal effects were significant (*P* < 0.001) for only DTA, while nonmaternal effects were significant for DTA, GY, and AUDPC (**Table 2**). Furthermore, the GCA × E interaction was significant for both agronomic and disease parameters, while the SCA × E interaction was significant for only DTA, GY, and MLN1. The reciprocal × E interaction was significant for DTA, MLN3 and AUDPC, while the maternal × E interaction was significant for DTA, MLN disease resistance parameters, and AUDPC. The GCA: SCA ratio varied for the agronomic traits and ranged from 0.65 to 0.85 for GY and agronomic traits, and from 0.70 to 0.73 for the MLN disease resistance parameters.

**Table 2:** Mean squares from combined analysis (Griffing’s Method 3 Model 1) for agronomic traits and MLN disease resistance parameters in a 14-parent diallel evaluated under artificial MLN inoculation at Naivasha over 3 years (2020−2022).

Source of variation	df	DTA	EPP	GY	MLN1	MLN2	MLN3	MLN4	AUDPC
Environment (E)	3	5643.69***	7.24***	246.55***	19.22***	16.30***	27.94***	49.26***	9626.44***
Rep(E)	4	12.05***	1.16***	0.51	0.04	0.42***	0.58***	0.45***	132.19***
Genotypes (G)	181	47.27***	0.84***	10.05***	3.47***	5.28***	6.32***	6.71***	2351.17***
GCA	13	351.74***	5.87***	64.02***	25.36***	39.33***	50.05***	53.22***	18188.86***
SCA	77	20.94***	0.98***	11.83***	3.73***	5.61***	6.26***	6.71***	2427.19***
Reciprocal	91	11.39***	0.22	0.97***	0.17	0.19	0.23	0.22	59.87*
Maternal (M)	13	11.80***	0.17	0.83	0.13	0.20	0.18	0.18	58.26
Nonmaternal (NM)	78	11.32***	0.23	1.00***	0.18	0.19	0.24	0.22	60.13*
G × E	542	7.02***	0.29	0.92***	0.28***	0.29***	0.38***	0.40***	104.57***
GCA × E	39	3218.49***	0.77***	4.05***	0.69***	0.72***	0.46***	0.76***	190.28***
SCA × E	231	807.56***	0.31	0.89***	0.22**	0.17	0.20	0.22	41.89
Reciprocal × E	273	4.86**	0.25	0.64	0.18	0.16	0.22*	0.22	54.41*
M × E	39	433.99***	0.19	0.66	0.29**	0.34***	0.52***	0.60***	157.90***
NM × E	234	0.00	0.26	0.64	0.17	0.13	0.18	0.15	37.16
Error	230	3.41	0.26	0.59	0.15	0.15	0.18	0.18	43.11
GCA: SCA ratio		0.85	0.68	0.65	0.70	0.70	0.73	0.73	0.72

*, **, and *** indicate significance at the 0.05, 0.01, and 0.001 levels, respectively.

DTA, days to anthesis; EPP, ears per plant; GY, grain yield; MLN1, MLN2, MLN3, and MLN4, maize lethal necrosis disease rating at 21, 28, 35 and 42 days after inoculation, respectively; AUDPC, area under disease progress curve.

### 3.2 ANOVA under rainfed conditions

The combined ANOVA under rainfed conditions revealed significant (*P* < 0.001) environment (E), genotype (G), and G × E interaction mean squares for all traits except the EPP genotype mean squares (**Table 3**). The results revealed that both GCA and SCA mean squares were significant (*P* < 0.001) for all the traits except EPP, which showed only significant GCA mean squares. The reciprocal differences between the F_1_ hybrids and their crosses were significant for DTA, EPP, and GY. Both maternal and nonmaternal effects were significant for DTA, and significant maternal effects were recorded for TLB. Nonmaternal effects were significant for GY and EPP. The GCA × E interaction was significant for all traits, while SCA × E interaction was significant for EPP and GY. The reciprocal × E and maternal × E interactions were significant for flowering traits DTA and DTS. The GCA: SCA ratio ranged from 0.50 to 0.88 for the agronomic traits and was 0.94 for TLB.

**Table 3:** Mean squares from combined analysis (Griffing’s Method 3 Model 1) for agronomic traits and Turcicum leaf blight in a 14-parent diallel evaluated under rainfed conditions at Kakamega, 2020−2022.

Source of variation	df	DTA	DTS	EPP	GY	TLB
Environment (E)	2	1684.04***	2170.82***	2.02**	1699.85***	1158.48***
Rep(E)	3	25.26***	34.34***	0.10	11.80***	0.77***
Genotypes (G)	181	36.08***	40.49***	0.06	6.86***	5.10***
GCA	13	369.37***	372.89***	0.14***	34.47***	62.96***
SCA	77	17.21***	25.28***	0.05	6.89***	1.38***
Reciprocal	91	5.51***	7.08	0.06*	3.04*	0.74
Maternal (M)	13	8.05***	9.14	0.06	2.99	1.36**
Nonmaternal (NM)	78	5.08***	6.74	0.06*	3.04*	0.63
G × E	357	5.91***	8.29***	0.06*	3.29**	0.95**
GCA × E	26	296.39***	295.15***	0.14***	5.96***	6.98***
SCA × E	154	0.0	0.0	0.07***	3.13**	0.53
Reciprocal × E	182	4.88***	7.78**	0.05	2.69	0.59
M × E	26	142.79***	155.74***	0.07*	2.70	0.84
NM × E	156	0.0	0.0	0.04	2.69	0.55
Error	168	2.65	5.43	0.04	2.35	0.58
GCA: SCA ratio		0.88	0.83	0.50	0.65	0.94

*, **, and *** indicate significance at the 0.05, 0.01, and 0.001 levels, respectively.

AUDPC, Area under disease progress curve; DTA, days to anthesis; DTS, days to silking; EPP, ears per plant; GY, grain yield; TLB, Turcicum leaf blight.

### 3.3 Performance of hybrids

The mean GY of the F_1_ hybrids under artificial MLN inoculation was 2.0 t ha^-1^, with a range of 0.4 to 6.4 t ha^-1^, while the GY of the commercial check hybrids ranged from 0.9 to 3.4 t ha^-1^ (**Table 4**). The range of the second disease rating taken 28 days after inoculation (MLN2) was 2.5 to 6.3, while the fourth disease rating taken 42 days after inoculation (MLN4) was 3.4 to 7.7. The variance due to genetic effects was 2.5 times greater than the residual variance for GY. For the MLN disease scores, the variance due to genetic effects was 3.9 to 4.2 times greater than the residual variance. Broad-sense heritability estimates were high for most traits (0.89−0.95) and moderate for EPP (0.67). The hybrid with the highest yield under artificial MLN inoculation was the reciprocal cross P6 × P2 (6.4 t ha^-1^), which also had the lowest disease scores for MLN3 (2.8) and MLN4 (3.4), and lowest AUDPC (55.4) (**Supplementary Table S1**). Grain yield was negatively correlated with all four MLN disease ratings (*r* = -0.80 to -0.86, *P* < 0.001). Under rainfed conditions, the mean GY was 4.0 t ha^-1^ and 4.8 t ha^-1^ for the F_1_ hybrids and commercial check hybrids, respectively (**Table 4**). Overall, the GY ranged from 2.5 to 6.0 t ha^-1^ under rainfed conditions. The mean number of DTA was greater at Naivasha (2086 m asl) than at Kakamega (1585 m asl), possibly due to the higher elevation and cooler environment at Naivasha. The TLB disease rating ranged from 2.4 to 5.8. Broad-sense heritability was moderate for GY (0.56) and high for agronomic traits and TLB (0.82−0.86), except for EPP. The top hybrid for GY under rainfed conditions at Kakamega was P12 × P2 (6.0 t ha^-1^), with a TLB score of 2.9 (**Supplementary Table S2**).

**Table 4:** Summary statistics, variance component and heritability estimates for agronomic traits, MLN disease resistance parameters, and area under disease progress curve (AUDPC) of 182 maize hybrids and four commercial hybrid checks evaluated under artificial inoculation with MLN at Naivasha, and under rainfed conditions at Kakamega for three seasons, 2020−2022.

Trait	Unit	Mean	Range	LSD_0.05_	Genotypic variance	G × E variance	Residual variance	Heritability
Naivasha (artificial MLN inoculation)								
GY (F_1_ hybrids)	t ha^-1^	2.0	0.4−6.4	1.0	1.44	0.10	0.77	0.92
GY (Checks)		2.2	0.9−3.4					
DTA	days	92	84−97	2.5	6.38	1.34	3.38	0.89
EPP	no.	0.9	0.4−1.9	0.4	0.07	0.01	0.26	0.67
MLN1	1−9	3.5	2.1−4.9	0.5	0.40	0.05	0.16	0.92
MLN2	1−9	4.4	2.5−6.3	0.5	0.63	0.06	0.16	0.95
MLN3	1−9	5.0	2.8−7.3	0.6	0.75	0.08	0.18	0.94
MLN4 (F1 hybrids)	1−9	5.4	3.4−7.7	0.6	0.80	0.10	0.19	0.94
MLN4 (Checks)		5.8	4.8−6.8					
AUDPC (F_1_ hybrids)		193.9	110.7−279.2	19.9	1133.51	111.50	180.99	0.96
AUDPC (Checks)		202.7	173.7−245.2					
Kakamega (rainfed)								
GY (F_1_ hybrids)	t ha^-1^	4.0	2.5−6.0	1.5	0.60	0.41	2.07	0.56
GY (Checks)		4.8	4.1−5.4					
DTA	days	80	75−86	2.5	5.45	0.93	2.97	0.87
DTS	days	80	75−85	3.0	5.77	0.82	5.65	0.83
EPP	no.	1.1	0.8−1.3	-	0.00	0.01	0.04	0.04
TLB (F_1_ hybrids)	1−9	4.1	2.4−5.8	1.0	0.67	0.17	0.53	0.82
TLB (Checks)		4.1	2.7−4.6					

AUDPC, area under disease progress curve; DTA, days to anthesis; DTS, days to silking; EPP, ears per plant; GY, grain yield; MLN1, MLN2, MLN3 and MLN4, maize lethal necrosis disease rating at 21, 28, 35 and 42 days after inoculation, respectively; TLB, Turcicum leaf blight.

### 3.4 ANOVA for inbred lines and *per se* performance

**Figure 1:**
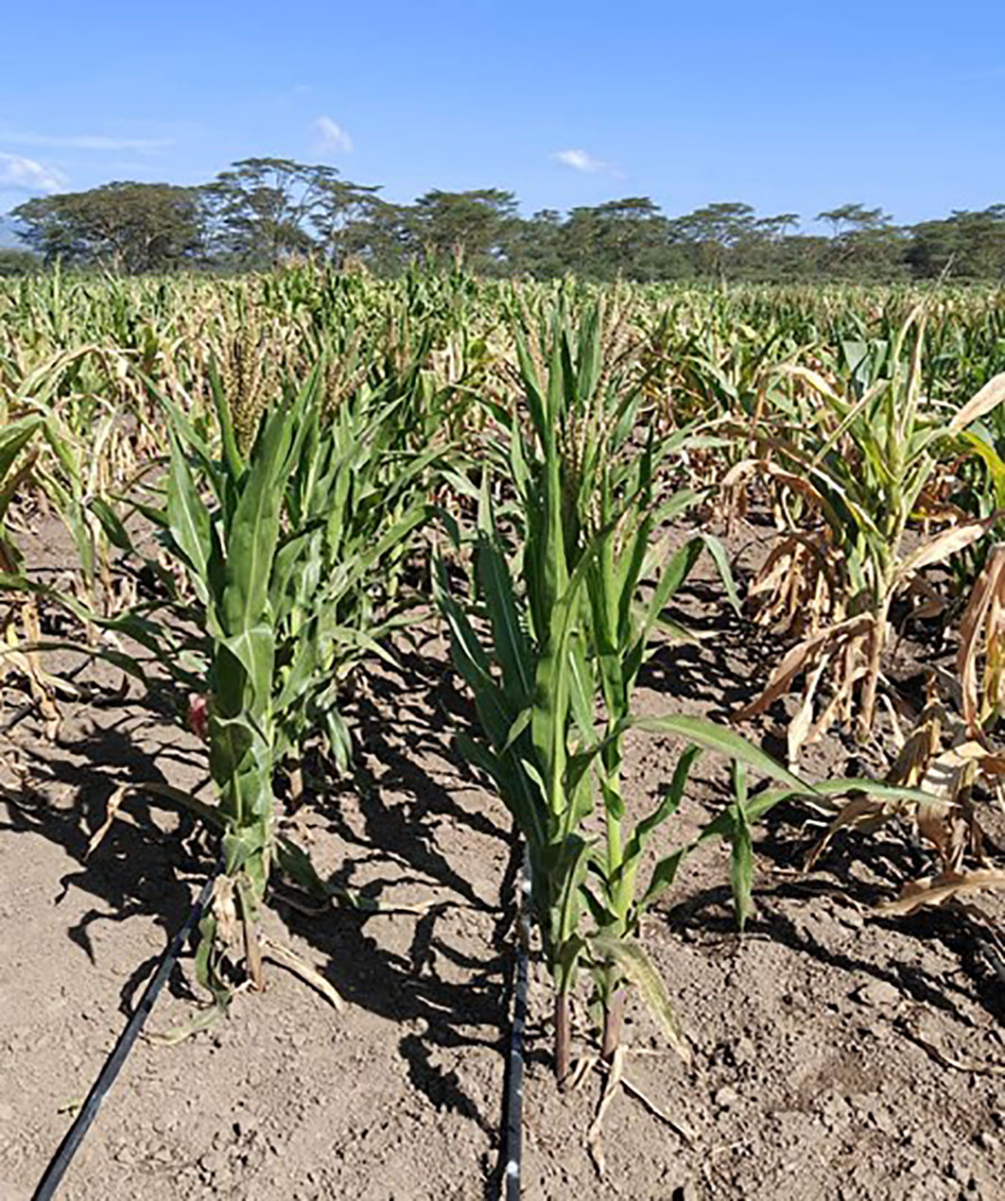
Inbred line CKL181281 under artificial MLN inoculation at the KALRO-CIMMYT MLN Screening Facility at Naivasha. This line was the most resistant in this study and was rated 2.1 on a scale of 1˗9. On the right is an MLN susceptible inbred line.

Combined ANOVA revealed significant (*P* < 0.001) genotype (G), and G × E interaction mean squares for MLN disease ratings and AUDPC under artificial MLN inoculation while significant genotype mean squares were recorded for TLB under rainfed conditions (**Supplementary Table S3**). The MLN disease rating ranged from 2.1 to 6.0, 2.1 to 7.4, 2.1 to 8.5, and 2.1 to 8.6 for MLN1, MLN2, MLN3 and MLN4, respectively. The most MLN resistant line with a score of 2.1 was CKL181281 (**Table 5**; **Figure 1**). The TLB disease rating for these lines ranged from 2.4 to 4.8. Broad-sense heritability was high for the MLN disease ratings, AUDPC and TLB (0.87−0.96) and moderate for DTA.

**Table 5:** General combining ability (GCA) estimates for maize lethal necrosis disease ratings, AUDPC, and agronomic traits, and *per se* performance of 14 maize inbred lines under artificial MLN inoculation at Naivasha over 3 years (2020−2022).

Line	Name	MLN1	MLN2	MLN3	MLN4	AUDPC	DTA (days)	EPP (No)	GY (t ha^-1^)	MLN3	MLN4	AUDPC
		(1−9)								(1−9)		
1	CKL18912	0.27***	0.28***	0.45***	0.56***	8.01***	1.88***	-0.16**	-0.24***	4.8	6.0	93.4
2	CKL181281	-0.49***	-0.57***	-0.53***	-0.45***	-11.05***	-2.34***	0.17***	0.80***	2.1	2.1	43.2
3	CKL181379	-0.29***	-0.44***	-0.39***	-0.35***	-8.06***	-0.58***	0.04	0.09	3.4	3.7	65.9
4	CKL181847	-0.13***	-0.19***	-0.04	0.07	-1.82***	1.09***	0.04	0.01	3.7	4.4	72.1
5	CKL182037	-0.25***	-0.35***	-0.36***	-0.24***	-6.66***	-1.77***	0.07	0.69***	3.4	3.7	65.4
6	CKL176616	-0.51***	-0.62***	-0.80***	-0.81***	-14.54***	0.42**	0.25***	1.23***	2.9	3.3	58.6
7	CKL175951	0.01	-0.01	-0.02	-0.16***	-0.74	0.65***	0.01	-0.29***	5.2	5.7	103.6
8	CKL175755	-0.13***	-0.23***	-0.55***	-0.63***	-8.14***	0.73***	0.06	-0.08	4.7	5.0	95.0
9	CKL175798	-0.07	-0.05	-0.03	-0.19***	-1.45*	-0.21	0.07	0.26***	5.1	5.4	98.8
10	CKL176082	-0.05	-0.01	-0.02	-0.13**	-0.86	1.44***	0.02	-0.33***	5.5	6.2	105.0
11	CKDHL120918	0.04	0.24***	0.28***	0.29***	4.74***	-1.89***	0.09*	0.02	4.3	5.0	79.9
12	CML585	0.81***	0.93***	1.07***	1.11***	20.69***	-0.88***	-0.48***	-1.02***	8.5	8.6	163.3
13	CKL14546	0.31***	0.47***	0.41***	0.41***	8.65***	-0.15	-0.05	-0.37***	6.0	6.6	111.0
14	CML444	0.47***	0.58***	0.53***	0.52***	11.22***	1.59***	-0.12***	-0.66***	6.7	6.7	123.4
***15***	***KS23-6*** ^†^									***3.9***	***4.2***	***74.3***
SE/LSD_(0.05)_^§^		0.03	0.03	0.03	0.03	0.46	0.13	0.04	0.05	0.8	1.0	13.5

*, **, and *** indicate significance at the 0.05, 0.01, and 0.001 levels, respectively.

^†^KS23-6, an MLN resistant donor line was used as a check in the line trial.

^§^SE of GCA effects; LSD for per se performance.

AUDPC, area under disease progress curve; DTA, days to anthesis; EPP, ears per plant; GY, grain yield; MLN1, MLN2, MLN3 and MLN4, maize lethal necrosis disease rating at 21, 28, 35 and 42 days after inoculation, respectively.

### 3.5 Estimates of GCA and SCA effects

The GCA effects varied between parents for the different traits. Inbred lines CKL181281, CKL181379, CKL182037, CKL176616, and CKL175755 had significant negative GCA effects for all four MLN disease resistance parameters and AUDPC, and therefore contributed to MLN resistance in their hybrids (**Table 5**). Inbred line CKL176616 had the largest significant negative GCA effect for both MLN4 (-0.81, *P* < 0.001) and AUDPC (-14.54, *P* < 0.001), followed by CKL175755 for MLN4 (-0.63, *P* < 0.001). Another inbred line, CKL181847, had significant negative GCA effects for two of the four MLN disease ratings (MLN1 and MLN2) and AUDPC. Three inbred lines (CKL175951, CKL175798, and CKL176082) had significant negative GCA effects for the fourth MLN disease rating. In contrast, five inbred lines showed significant positive GCA effects for three MLN ratings (MLN2−MLN3) and AUDPC, suggesting that these lines contributed to MLN susceptibility in their hybrids. Inbred lines CKL181281, CKL182037, CKL176616, and CKL175798 exhibited significant positive GCA effects for GY under artificial MLN inoculation. We computed the nonparametric Spearman’s rank correlation coefficient between the GCA effects for the four MLN disease resistance parameters, and the results revealed very strong correlations among the scores (*r_s_* = 0.93−0.98, *P* < 0.001). In terms of SCA effects, the results showed that nearly a similar number of hybrids had significant negative SCA effects on the second (35), third (37), and fourth (35) MLN disease rating (**Supplementary Tables S4**, **S5**). Several hybrid combinations, such as CKL181281 × CKL182037, CKL18912 × CKL181281, CKL18912 × CKL182037, and CKL181379 × CKL182037 consistently exhibited negative significant SCA effects for MLN2, MLN3, and MLN4, suggesting their potential for MLN disease resistance. For GY, 31 hybrids (33%) exhibited significant positive SCA effects under artificial MLN inoculation. Twenty-nine hybrids had significant negative SCA effects for the fourth MLN disease rating and significant positive SCA effects for GY. The top-yielding hybrids were between parents with desirable GCA effects for MLN resistance and GY. For example, the top two hybrids (P6 [CKL176616] × P2 [CKL181281], SCA = 2.0 t ha^-1^ and P6 [CKL176616] × P5 [CKL182037], SCA = 1.2 t ha^-1^) were between lines with significant GCA effects for both traits. Furthermore, P6 (CKL176616), P5 (CKL182037) and P2 (CKL181281) were the parents of 18 of the top 25 hybrids in terms of GY performance under MLN inoculation.

Under rainfed conditions, the inbred lines CKL18912, CKL175951, CKL175755, CKL176082, and CML444 had significant positive GCA effects for both maturity parameters (**Table 6**). Five inbred lines (CKL181281, CKL182037, CKDHL120918, CML585, and CKL14546) exhibited significant positive GCA effects for GY under rainfed conditions. Six inbred lines showed significant desirable GCA effects for TLB. Inbred lines CKL181281 and CKL182037, which showed significant and desirable GCA effects for MLN resistance and GY under both artificial MLN inoculation and rainfed conditions, also exhibited significant negative GCA effects for TLB. The results indicated that 10 hybrids had significant positive SCA effects for GY, while 10 hybrids had significant negative SCA effects for TLB (**Supplementary Table S6**). Under rainfed conditions, either P2 (CKL181281) or P5 (CKL182037) was the parent of 11 of the top 25 hybrids for GY.

**Table 6:** General combining ability (GCA) estimates for agronomic traits, grain yield, and TLB, and *per se* performance of 14 maize inbred lines under rainfed conditions in Kakamega over 3 years (2020−2022).

Line	Name	GCA Effects					*Per se*
		DTA (days)	DTS (days)	EPP (No)	GY (t ha^-1^)	TLB (1-9)	TLB (1-9)
1	CKL18912	1.35***	1.89***	-0.02	-0.44**	0.21**	3.4
2	CKL181281	-1.14***	-0.37	0.03	0.62***	-0.87***	2.4
3	CKL181379	-0.38**	-0.49*	-0.01	-0.66***	-0.93***	2.4
4	CKL181847	0.24	1.20***	0.01	-0.23	0.17**	2.9
5	CKL182037	-0.60***	-0.23	-0.03	0.29*	-0.51***	2.7
6	CKL176616	-0.20	-0.32	-0.04*	-0.78***	1.26***	4.8
7	CKL175951	1.19***	0.45*	0.02	0.02	-0.01	2.9
8	CKL175755	2.18***	1.90***	0.02	-0.56***	1.00***	3.9
9	CKL175798	-0.97***	-1.77***	-0.00	0.07	0.37***	4.2
10	CKL176082	1.99***	1.96***	0.06**	-0.03	0.34***	3.6
11	CKDHL120918	-2.22***	-3.14***	-0.04*	0.14*	0.31***	3.6
12	CML585	-2.54***	-1.61***	-0.05*	0.62***	-0.79***	3.3
13	CKL14546	-1.27***	-1.27***	0.04*	0.90***	-0.27***	2.5
14	CML444	2.36***	1.80***	-0.01	0.03	-0.29***	3.3
***15***	***KS23-6*** ^†^						***3.6***
SE/LSD_(0.05)_^§^		0.13	0.19	0.02	0.12	0.06	0.8

*, **, and *** indicate significance at the 0.05, 0.01, and 0.001 levels, respectively.

^†^KS23-6, an MLN resistant donor line was used as a check in the line trial.

^§^SE of GCA effects; LSD for *per se* performance.

DTA, days to anthesis; DTS, days to silking; GY, grain yield; EPP, ears per plant; TLB, Turcicum leaf blight.

### 3.6 Genetic parameters of MLN resistance

The quantitative genetic parameters for MLN disease resistance were studied using Hayman (1954a) diallel analysis model. The ANOVA revealed that both additive (a) and dominant (b) gene effects were significant in the control of MLN resistance, but additive gene effects were more important in the inheritance of MLN resistance (**Table 7**). Furthermore, the analysis revealed that neither maternal (c) nor reciprocal effects (d) were significant for the four MLN disease resistance parameters, indicating that there were no significant differences between reciprocal crosses. This result is consistent with Griffing’s diallel analysis for the same traits (**Table 2**). The results show that there was significant directional dominance (b_1_). A comparison of the hybrid and parental means shows that dominance was for susceptibility to MLN. The interactions a × E, b × E, c × E, and d × E were significant for the four MLN disease resistance parameters, suggesting that gene effects were influenced by the environment. The genetic parameter estimates for the four MLN disease ratings are presented in **Table 8**. The additive and dominance variance components were both significant for the four MLN disease resistance parameters, but the additive variance component was of greater magnitude. This suggested a greater role of additive gene action in MLN resistance. Gene frequency asymmetry was detected but was less important for all four disease parameters (H_2_/4H_1_ < 0.20). The mean degree of dominance was 0.89 for MLN1, 0.93 for MLN2, 0.85 for MLN3, and 0.89 for MLN4, indicating incomplete dominance for MLN resistance. The minimum number of groups of genes for MLN resistance was estimated to be 3.53, 3.82, 3.63, and 3.31 for MLN1, MLN2, MLN3, and MLN4, respectively. The correlation coefficient between *W_r_* + *V_r_* and *Y_r_* (parental mean) was negative (−0.64 to −0.59) for the MLN parameters, suggesting that the dominant genes increased susceptibility to MLN. Narrow-sense heritability estimates ranged from 0.52 to 0.56 for the four MLN disease resistance parameters.

**Table 7:** Mean squares from ANOVA of the 14-parent diallel for MLN disease resistance parameters under artificial inoculation with MLN based on Hayman’s (1954a) method.

Item	Effect	df	MLN1	MLN2	MLN3	MLN4
a	Additivity	13	32.78***	50.59***	66.48***	68.84***
b	Dominance	91	3.56***	5.56***	6.42***	7.06***
b1	Directional dominance	1	3.01***	8.92***	8.28***	7.72***
b2	Gene distribution	13	2.59***	4.98***	7.18***	9.08***
b3	Residual dominance	77	3.73***	5.61***	6.26***	6.71***
c	Maternal	13	0.13ns^†^	0.20ns	0.18ns	0.18ns
d	Reciprocal	78	0.18ns	0.19ns	0.24ns	0.23ns
a × Environment (E)		39	0.75***	0.65***	0.49***	0.71***
b × E		273	0.38***	0.33***	0.34***	0.46***
b1 × E		3	10.77***	10.96***	10.38***	12.71***
b2 × E		39	0.56***	0.43***	0.38***	0.89***
b3 × E		231	0.22***	0.17ns	0.20ns	0.24ns
c × E		39	0.29***	0.34***	0.52***	0.60***
d × E		234	0.17***	0.13***	0.18***	0.16***
Error		774	0.17	0.17	0.22	0.23

*** indicates significance at the 0.001 level.

^†^ns, not significant.

MLN1, MLN2, MLN3, and MLN4 are maize lethal necrosis disease ratings at 21, 28, 35, and 42 days after inoculation, respectively.

**Table 8:** Mean genetic parameters for MLN disease resistance parameters based on diallel analysis of 14 inbred lines evaluated under artificial inoculation with MLN at Naivasha over 3 years (2020−2022).

Genetic parameter	MLN1	MLN2	MLN3	MLN4
Additive variance component (D)	1.17** ± 0.11	1.94** ± 0.14	2.84** ± 0.24	2.94** ± 0.31
Dominant variance component (H_1_)	0.93** ± 0.20	1.68** ± 0.26	2.06** ± 0.46	2.32** ± 0.60
Dominant variance component (H_2_)	0.72** ± 0.16	1.22** ± 0.22	1.39** ± 0.38	1.52** ± 0.49
Dominant effect component (h^2^)	0.08 ± 0.11	0.27 ± 0.14	0.25 ± 0.25	0.20 ± 0.32
Relative frequency of dominant and recessive alleles (F)	0.80** ± 0.24	1.52** ± 0.31	2.34** ± 0.55	2.52** ± 0.70
Environmental variation (E)	0.08 ± 0.03	0.08 ± 0.04	0.11 ± 0.06	0.11 ± 0.08
Mean degree of dominance (√H_1_/D)	0.89	0.93	0.85	0.89
Minimum number of groups of genes	3.53	3.82	3.63	3.31
Correlation (*r*) between Wr+Vr and Yr	-0.59	-0.61	-0.59	-0.64
Broad-sense heritability	0.85	0.90	0.89	0.89
Narrow-sense heritability	0.52	0.53	0.56	0.55

** indicates significance at the 0.01 level.

MLN1, MLN2, MLN3, and MLN4 are maize lethal necrosis disease ratings at 21, 28, 35, and 42 days after inoculation, respectively.

**Figure 2:**
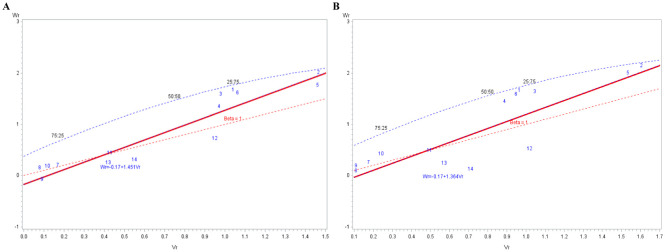
The relationship between the variance of the F_1_ for each parental line (*Vr*) and their covariance with the non-recurrent parent (*Wr*) for the MLN3 **(A)** and MLN4 **(B)** disease severity scores across four seasons, 2020−2022.

The variance (*V_r_*) and covariance (*W_r_*) estimates for the third and fourth MLN disease rating (when full expression of the response of lines to MLN was best observed) were used for regression analysis. The slope of the regression line was >1.0, suggesting adequacy of the model (**Figure 2**). The lines were spread along the regression line, which suggested diversity among the lines for MLN resistance. Based on the *W_r_-V_r_* plot, the inbred lines used in this study could be grouped into four groups. Group 1 included lines 7 (CKL175951), 8 (CKL175755), 9 (CKL175798), and 10 (CKL176082) which had more dominant alleles. Group 2 included lines 11 (CKDHL120918), 13 (CKL14546), and 14 (CML444), which had slightly more recessive alleles than did the lines in group 1. Group 3 was composed of lines 1 (CKL18912), 3 (CKL181379), 4 (CKL181847), and 6 (CKL176616), which had more recessive than dominant alleles (75:25). This group of lines showed negative and significant GCA effects for MLN disease resistance parameters and AUDPC, except for line 1 (CKL18912). Inbred lines 2 (CKL181281) and 5 (CKL182037) formed the fourth group and contained the most recessive alleles for MLN resistance.

## 4 Discussion

To effectively combat the spread of MLN in SSA, breeding for resistant varieties coupled with other strategies like clean seed as part of an integrated disease management strategy is important. The development of MLN resistant parental inbred lines with favorable alleles for key agronomic and adaptive traits is critical for the identification of high-yielding adapted MLN-resistant maize varieties. Our objective was to understand the genetics of resistance to MLN and other key traits in the late maturity maize germplasm, information that can be utilized in designing a breeding strategy for MLN resistance. The lines used in this study differed in terms of their source germplasm (drought tolerance, adaptation, and MLN resistance), and selection history with some lines developed through pedigree breeding and others developed through the DH technique. In the present study, highly significant genotypic differences for MLN disease resistance parameters and agronomic traits under artificial inoculation conditions were revealed. Highly significant genotypic differences were detected for all traits except EPP under rainfed conditions. These findings indicate that there was sufficient genetic variability for most of the traits studied in this set of germplasm, implying that progress from selection and ultimately genetic gain can be made in breeding for MLN resistance and other adaptive traits. Access to diverse germplasm resources has enabled CIMMYT to make progress in the development of MLN-resistant germplasm to combat the disease in Eastern Africa (Prasanna et al., 2020).

The broad-sense heritability for MLN disease resistance parameters was high (*H* ^2^ = 0.92–0.95) in this study which suggests that a greater proportion of the observed phenotypic variation in this germplasm was due to genetic variability, and that breeding for MLN resistance can lead to considerably high genetic gains from selection for these traits. This conclusion is supported by the moderately high narrow-sense heritability (*h* ^2^ = 0.52–0.56) for MLN disease resistance parameters which suggested that more than 50% of the genetic control was attributed to additive genetic effects. The implication is that the resistance of this germplasm to MLN can be improved relatively rapidly. There are no published reports on narrow-sense heritability for MLN disease resistance parameters to compare our results with. High broad-sense heritability estimates for virus diseases of maize have been previously reported, e.g., for MLN (Beyene et al., 2017), MCMV (Jones et al., 2018), and SCMV (Pokorny and Porubova, 2006).

The genetics of virus diseases have been investigated in tropical maize using a variety of intermediate maturity maize germplasm (Mutengwa et al., 2012; Nyaligwa et al., 2017; Beyene et al., 2017; Nyaga et al., 2020). In our study, we used maize in the late-maturity category to investigate the combining abilities and reciprocal effects in this germplasm. The results of this study revealed a greater contribution of GCA variance than of SCA variance for all traits under artificial MLN inoculation and for agronomic and disease traits under rainfed conditions. This suggests a preponderance of additive genetic effects in the inheritance of these traits in this germplasm. This result is consistent with findings for MLN in a study under artificial inoculation (Beyene et al., 2017), for maize streak virus (Mutengwa et al., 2012; Kim et al., 1989) and for maize dwarf mosaic virus (Loesch and Zuber, 1972; Rosenkranz and Scott, 1987). Both additive and dominant genes have been reported to condition virus resistance in maize (Naidu and Josephson, 1976; Zambrano et al., 2014). With a predominance of GCA over SCA, early testing may be more effective, and promising hybrids can be identified and selected mainly based on the prediction from GCA effects and the most resistant hybrids can be obtained by crossing the parents with the highest GCA (Baker, 1978; Makumbi et al., 2011). To improve both MLN resistance and grain yield potential, breeders can select lines with significant negative GCA effects for MLN resistance, significant positive GCA effects for grain yield, and significant negative GCA effects for foliar diseases to create new hybrid combinations for testing in the relevant target environments. Performance of hybrids can also be predicted based on the performance of single crosses (e.g. Zuber et al., 1973). Recurrent selection that emphasizes GCA can be an effective strategy to improve MLN resistance in this germplasm, as has been recommended for other virus diseases of maize (Josephson and Naidu, 1971; Kim et al., 1989; Mutengwa et al., 2012). With the implementation of forward breeding for MLN and other diseases such as MSV, the identification of MLN resistant lines that possess other adaptive traits for hybrid development should lead to faster genetic gains.

The consistency observed in the GCA effects across the four time points suggests rating genotypes for MLN resistance on multiple dates may not be necessary. Evaluating genotypes at 35 days (MLN3) and 42 (MLN4) days after inoculation should be sufficient for accurate assessment of resistance. The results revealed that five inbred lines had consistently significant desirable (negative for reduced disease) GCA effects for the four MLN resistance scores and AUDPC. This suggested that these lines have favorable alleles for resistance to MLN and can slow disease progression. Inbred line 6 (CKL176616) which had the largest desirable GCA effects for the four MLN resistance ratings derived its resistance alleles from KS23-5 unlike three of the four lines (CKL181281, CKL181379, and CKL182037) whose resistance was from KS23-6. Interestingly, among the five inbred lines, inbred line CKL175755 is not known to have any pedigree breeding history of resistance to viruses. Two other lines (CKL175798 and CKL176082), also without a background of virus resistance showed significant negative GCA effects for MLN4. Detailed studies (e.g., Jones et al., 2018) under artificial MLN, MCMV, and SCMV inoculation in net houses are needed to confirm the response of these lines as they may offer an additional source of alleles for MLN resistance. To date, only lines KS23-5 and KS23-6 have validated QTLs for MLN resistance (Murithi et al., 2021; Awata et al., 2021), but QTLs for resistance to MCMV, one of the two viruses that cause MLN have been mapped in other lines (Jones et al., 2018). In our study, the inbred line CKDHL120918 had significant positive GCA effects for MLN but this line was reported to have significant negative GCA effects for MLN in an earlier study (Beyene et al., 2017). The differences in GCA effects of this line between the two studies could be due to variation in the diallel method used, as different methods can impact GCA estimates (Fan et al., 2014). We used Method 3 in this study, while Beyene et al. (2017) used Method 4 of Griffing (1956). Four inbred lines (CKL181281, CKL182037, CKL176616, and CKL175798) out of the five that had significant negative GCA effects for all MLN disease resistance parameters and AUDPC also expressed significant positive GCA effects for GY under artificial MLN inoculation. These findings demonstrate progress made in developing lines with favorable alleles for MLN-resistance (reduced disease susceptibility) while also combining beneficial alleles for GY under both MLN-infected and rainfed conditions. Furthermore, two of the four lines (CKL181281 and CKL182037) had significant positive GCA effects for GY and significant negative GCA effects for *Turcicum* leaf blight under rainfed conditions. These two lines with desirable GCA effects for GY and disease resistance across different conditions have the potential as inbred line testers for the MLN breeding program if the right testing strategies (e.g. Castellanos et al., 1998; Pswarayi and Vivek, 2008) are used to confirm their suitability as testers. Suitable testers must correctly classify and discriminate efficiently among test entries (Rawlings and Thompson, 1962).

The use of reciprocal crosses provides a quantitative method to assess the contribution of maternal effects in the inheritance of a trait. When reciprocal differences are strong, parental inbred performance affects the choice of a female parent in a hybrid (Mann and Pollmer, 1981). In this study, reciprocal and maternal effects were not significant for MLN disease resistance parameters, indicating that the disease parameters recorded were not influenced by maternal effects or cytoplasmic inheritance. This suggests that a line that is resistant to MLN can be used either as a female or male parent in a hybrid combination, although other traits such as seed producibility and pollen production must be considered. According to Roach and Wullf (1987), reciprocal crosses have similar nuclear genetic contributions, and any divergence in the performance of reciprocal pairs is due to a maternal or perhaps a paternal effect. The absence of maternal influence on the MLN disease resistance parameters signifies the predominance of additive gene action for MLN resistance since maternal effects are assumed to result from nonadditive gene action. Furthermore, maternal effects can potentially decrease the accuracy of genetic studies. Both cytoplasmic and nuclear maternal genetic effects may increase the observed genetic variance, but if the trait is fully controlled by maternal factors, they could curtail the response to selection (Roach and Wullf, 1987). Therefore, lack of maternal effects in this study suggests that the response to selection for MLN resistance will be minimally impacted. Our results indicated significant reciprocal effects for several agronomic traits including DTA and GY under MLN inoculation, and DTA, EPP, and GY under rainfed conditions. These findings were consistent with other reports of significant reciprocal effects for GY in maize (Ordás et al., 2008; Kagoda et al., 2011). However, our results contrast with those of Jumbo and Carena (2008) and Machida et al. (2010) who reported no significant effects for GY.

The development of multiple stress tolerant maize germplasm is an objective of many breeding programs in SSA, and this requires the selection of parental lines with suitable breeding values for the target traits. In SSA, stress tolerances including tolerance to foliar diseases (TLB and gray leaf spot), viral diseases (MSV and MLN) and low soil fertility and drought stress are required in certain combinations in hybrids for commercial production. The results showed that several lines had desirable GCA effects for a combination of some of the stresses. These lines should be tested in hybrid combinations under managed abiotic stress conditions mainly drought and low soil fertility following established protocols (e.g. Njeri et al., 2017; Makumbi et al., 2018b). Lines that have the highest breeding values across several stresses can be used as parents in biparental populations for development of new inbred lines. Further improvement of tropical germplasm using the new lines can be achieved with the application of modern tools and techniques such as DH, genomic selection, and marker-assisted selection with improvements in phenotyping methods to increase the rate of genetic gain and develop new multiple stress tolerant inbred lines (Cooper et al., 2014; Cobb et al., 2019; Prasanna et al., 2021; 2022).

The diallel analysis model of Hayman (1954a, b) provides estimates of several quantitative genetic parameters for the traits of interest. This method of analysis has been applied to the diallel analysis of quantitative traits alongside Griffing (1956) method to gain a deeper understanding of the inheritance of a trait beyond what a single method of diallel analysis can provide (e.g. Naidu and Josephson, 1976; Hamid et al., 1982; Betrán et al., 2003; Kagoda et al., 2011). The current study is the first to investigate the quantitative genetic parameters of MLN resistance using Hayman’s model. The results indicated that additive effects had a greater contribution in the inheritance of MLN resistance compared to dominance effects based on Hayman’s method, a result similar to that obtained using Griffing’s method for combining ability analysis. A study on the inheritance of *Helminthosporium* leaf spot in maize (Hamid et al., 1982) also reported congruence between results from Hayman’s and Griffing’s models for several disease resistance parameters. Our results revealed that the alleles controlling resistance or susceptibility to MLN were not equally distributed among the lines used in this study (H_2_ < H_1_). This was evident through the distribution of the parental arrays along the regression line on the *W_r_-V_r_* graph. The 14 parents were therefore unique with respect to the dominance and/or epistatic effects of the genes they possess (Allard, 1956). The *W_r_-V_r_* graphs for MLN3 and MLN4 were similar in the placement of the arrays along the regression line. The group of lines that were among the most resistant to MLN (P2 [CKL181281] and P5 [CKL182037]) were located far from the origin at the upper end of the regression line, which indicated that the alleles conditioning resistance in these lines were mostly recessive. These inbred lines had desirable GCA effects for MLN resistance and AUDPC. A group of four lines (P7, P8, P9, and P10) was located closer to the origin of the regression line, which indicated that this set of lines possessed more dominant alleles. Some of these lines, especially P8 and P9, showed significant negative GCA effects for MLN and AUDPC. This finding is of particular interest and necessitates further investigation to understand the genetic basis of resistance in these lines given that resistance to MLN has been reported to be recessive. The positioning of P8 and P9 with respect to other lines with significant negative GCA effects for MLN and exhibited resistance such as P2, P3, P5 and P6, was surprising. This may suggest that these lines exhibited similar phenotypes through different genetic mechanisms (Luckett, 1989). These lines should be tested under artificial inoculation with individual MCMV and SCMV isolates in a net house for better discrimination in terms of resistance to these viruses.

## 5 Conclusions

This study revealed that additive genetic effects contribute significantly to the inheritance of MLN in late maturity germplasm adapted to eastern Africa. Five inbred lines, three of which were derived from introgression of MLN resistance from KS23-5 and KS23-6 exhibited significant desirable GCA effects for MLN resistance and GY under artificial MLN conditions. Three of these lines also showed significant desirable GCA effects for GY under disease and rainfed conditions and have the potential to contribute to the development of multiple stress tolerant hybrids for the target product profile. We identified four inbred lines with desirable GCA effects for MLN resistance despite having no known breeding history of virus resistance. Detailed studies under artificial inoculation with individual viruses (MCMV and SCMV) and MLN should help to decipher the genetic basis of resistance to MLN in this group of lines. Reciprocal effects were of minor importance; therefore, breeding programs can use any MLN resistant inbred line as a female or male in hybrid combinations without a significant effect on the MLN response in the final product. The graphical method of analysis revealed the distribution of the lines in relation to the abundance of recessive or dominant alleles, and this information will be useful for selecting parents for biparental populations and hybrid development.

## Data Availability

The data sets used in this study have all been analyzed and summarized in the Supplementary Tables presented.
