# Supplementary material for: Inheritance of resistance to maize lethal necrosis in tropical maize inbred lines
Source: Front Plant Sci. 2025 Jan 9;15:1506139. doi: 10.3389/fpls.2024.1506139 (PMC11753913; doi:10.3389/fpls.2024.1506139)
Supplement: Supplementary file 1 [file Table1.docx]

SUPPLEMENTAL TABLE 1 Best linear unbiased predictions (BLUP) for agronomic traits, maize lethal necrosis (MLN) disease rating, and AUDPC of 186 maize hybrids evaluated under artificial MLN inoculation at Naivasha over 3 years (2020-2022).

Hybrid Parents GY (ha-1) DTS (days) EPP (No.) MLN1 (1‒9) MLN2 (1‒9) MLN3 (1‒9) MLN4 (1‒9) AUDPC

1 P1/P2 3.6 92 1.0 2.8 3.3 4.3 4.9 80.1

2 P2/P1 2.7 92 1.1 2.8 3.1 4.1 4.6 75.5

3 P1/P3 2.1 91 1.1 3.0 3.9 4.5 4.8 87.0

4 P3/P1 1.9 92 0.9 3.2 3.9 4.9 5.5 91.3

5 P1/P4 1.8 92 1.0 3.3 4.1 4.9 5.5 93.9

6 P4/P1 1.8 91 0.9 2.8 3.8 4.9 5.3 89.1

7 P1/P5 3.5 91 1.0 2.8 3.3 4.3 4.8 79.6

8 P5/P1 3.7 92 1.1 2.7 3.1 3.9 4.4 73.6

9 P1/P6 3.4 92 1.1 2.8 3.5 4.3 4.7 81.3

10 P6/P1 4.1 92 1.2 2.3 3.2 4.1 4.7 74.8

11 P1/P7 1.4 92 0.8 4.1 5.0 5.7 6.2 111.1

12 P7/P1 1.0 91 0.8 4.0 5.1 5.7 6.2 111.2

13 P1/P8 0.7 92 0.7 3.8 4.4 5.3 5.6 99.8

14 P8/P1 1.0 92 0.7 3.9 4.9 5.1 5.5 102.4

15 P1/P9 1.3 93 0.8 4.2 5.2 5.7 6.1 112.7

16 P9/P1 2.0 92 0.9 4.0 5.0 5.6 6.1 108.8

17 P1/P10 1.2 92 0.7 3.8 4.9 5.6 6.2 107.8

18 P10/P1 1.1 92 0.7 4.1 5.1 5.5 6.2 109.5

19 P1/P11 0.9 92 0.7 4.3 5.6 5.9 6.7 118.9

20 P11/P1 1.2 91 0.9 4.3 5.2 6.1 6.6 116.9

21 P1/P12 0.9 91 1.0 4.9 6.3 7.4 7.7 139.6

22 P12/P1 0.4 91 0.5 4.9 6.1 7.0 7.5 137.1

23 P1/P13 0.9 92 0.8 4.5 5.7 6.4 6.9 125.2

24 P13/P1 1.1 91 0.8 4.3 5.7 6.4 6.8 124.0

25 P1/P14 0.9 92 0.7 4.5 5.8 6.5 6.8 126.4

26 P14/P1 0.8 92 0.7 4.5 5.8 6.4 6.9 125.8

27 P2/P3 2.6 90 1.1 2.6 3.1 3.7 4.1 71.1

28 P3/P2 3.1 90 1.0 2.4 3.1 3.6 4.2 69.8

29 P2/P4 3.3 91 1.2 2.3 3.1 3.9 4.8 73.6

30 P4/P2 2.5 91 1.4 2.6 3.2 4.1 4.9 76.6

31 P2/P5 4.8 90 1.4 2.1 3.0 3.1 3.8 62.8

32 P5/P2 4.8 89 1.4 2.4 3.0 3.3 3.8 65.0

33 P2/P6 5.7 91 1.4 2.2 2.7 3.0 3.4 59.5

34 P6/P2 6.4 90 1.5 2.1 2.5 2.8 3.4 55.4

35 P2/P7 1.5 90 1.0 3.6 4.5 5.2 5.3 99.2

36 P7/P2 1.4 91 0.9 3.8 4.6 5.2 5.4 101.2

37 P2/P8 1.9 91 1.1 3.3 4.3 4.6 5.0 91.2

38 P8/P2 1.9 91 1.0 3.3 4.2 4.6 4.9 91.4

39 P2/P9 2.5 90 1.0 3.3 4.3 5.2 5.3 96.3

40 P9/P2 2.1 89 1.0 3.3 4.5 5.2 5.3 97.8

41 P2/P10 1.8 92 1.0 3.2 4.1 5.0 5.3 93.4

42 P10/P2 1.9 91 0.9 3.4 4.5 5.2 5.3 98.8

43 P2/P11 3.9 89 1.2 2.3 3.3 3.7 4.0 70.4

44 P11/P2 3.2 89 1.2 2.5 3.3 4.1 4.6 75.1

45 P2/P12 0.6 90 0.5 4.3 5.3 6.1 6.8 118.4

46 P12/P2 1.3 90 0.6 4.0 5 5.8 6.3 111.5

47 P2/P13 2.1 90 1.0 3.5 4.5 5.1 5.7 99.6

48 P13/P2 2.2 90 1.1 3.7 4.9 5.4 5.8 105.1

49 P2/P14 0.8 90 0.6 4.1 5.1 5.9 6.6 115.3

50 P14/P2 0.6 90 0.9 4.1 5.2 5.8 6.7 114.7

51 P3/P4 2.0 92 1.0 2.8 3.3 4.3 4.7 79.7

52 P4/P3 2.9 92 1.1 2.7 3.3 4.1 4.7 77.1

53 P3/P5 4.4 90 1.2 2.2 2.7 3.2 3.7 62.1

54 P5/P3 4.6 90 1.1 2.3 2.7 3.2 3.8 62.7

55 P3/P6 3.9 90 1.3 2.2 2.7 3.2 3.6 61.4

56 P6/P3 4.7 90 1.4 2.3 2.8 3.3 3.7 64.1

57 P3/P7 2.0 91 0.9 3.4 4.3 4.9 5.4 94.4

58 P7/P3 1.5 91 1.1 3.4 4.1 5.0 5.0 92.5

59 P3/P8 1.0 91 0.9 3.2 4.2 4.6 5.0 89.6

60 P8/P3 1.8 92 1.0 3.2 4.0 4.3 4.8 85.4

61 P3/P9 1.9 91 1.0 3.4 4.4 4.8 5.1 93.5

62 P9/P3 2.3 91 1.0 3.0 4.1 4.8 5.1 90.8

63 P3/P10 1.7 92 1.1 3.4 4.3 4.9 4.9 93.0

64 P10/P3 1.6 92 1.1 3.5 4.3 4.8 4.8 92.3

65 P3/P11 1.5 91 1.1 3.7 5.0 5.6 6 107.9

66 P11/P3 1.6 90 0.9 3.7 4.7 5.2 6 103.6

67 P3/P12 0.5 91 0.4 4.4 5.3 6.2 6.8 119.9

68 P12/P3 0.6 90 0.4 4.4 5.4 6.4 6.9 123.3

69 P3/P13 1.2 91 0.9 3.7 4.5 5.4 5.5 101.8

70 P13/P3 1.5 91 0.9 3.6 4.6 5.2 5.6 100.9

71 P3/P14 0.6 92 0.8 4.2 4.7 5.5 6.1 108.2

72 P14/P3 0.8 92 0.8 4.2 5.0 5.8 6.4 112.8

73 P4/P5 3.9 91 1.2 2.5 3.2 4.1 4.7 75.6

74 P5/P4 3.9 91 1.2 2.5 3.0 3.8 4.3 70.1

75 P4/P6 3.2 91 1.3 2.5 2.8 3.6 4.0 67.4

76 P6/P4 3.6 91 1.3 2.6 3.1 3.6 4.2 70.4

77 P4/P7 1.4 91 0.9 3.4 4.3 5.1 5.1 95.4

78 P7/P4 1.7 92 0.9 3.4 4.3 5.0 5.3 95.3

79 P4/P8 1.7 92 1.0 3.6 4.1 4.6 5.1 92.3

80 P8/P4 1.8 92 0.9 3.6 4.3 4.6 5.0 92.5

81 P4/P9 2.3 92 1.0 3.3 4.3 4.9 5.3 93.9

82 P9/P4 2.0 91 1.0 3.6 4.6 5.1 5.5 99.9

83 P4/P10 1.6 90 1.2 3.6 4.6 5.2 5.6 101.1

84 P10/P4 1.5 93 0.9 3.5 4.6 5.2 5.5 100.2

85 P4/P11 1.0 91 0.8 3.9 5.0 6.1 6.4 114.1

86 P11/P4 1.5 91 0.8 4.1 5.0 5.8 6.3 111.8

87 P4/P12 0.5 93 0.5 4.4 5.8 6.5 7.1 127.1

88 P12/P4 0.8 92 0.4 4.4 5.4 6.4 6.9 123.3

89 P4/P13 1.3 92 0.9 3.8 4.9 5.4 6.0 106.3

90 P13/P4 1.2 92 0.9 4.1 5.1 5.7 6.1 111.8

91 P4/P14 1.2 93 0.8 4.3 5.4 5.9 6.7 118.3

92 P14/P4 0.8 91 0.7 4.4 5.7 6.4 6.8 123.6

93 P5/P6 4.2 90 1.3 2.4 2.8 3.0 3.7 61.5

94 P6/P5 6.1 90 1.5 2.2 2.8 3.0 3.7 61.0

95 P5/P7 1.7 90 1.0 3.5 4.4 5.1 5.1 97.1

96 P7/P5 1.7 91 1.1 3.5 4.4 5.2 5.5 98.7

97 P5/P8 2.0 91 0.9 3.4 4.1 4.5 4.9 89.8

98 P8/P5 2.0 91 1.1 3.4 4.3 4.4 4.9 89.7

99 P5/P9 1.8 89 0.9 3.5 4.4 4.9 5.1 95.2

100 P9/P5 2.1 90 1.0 3.6 4.4 5.0 5.1 96.3

101 P5/P10 1.9 91 1.0 3.3 4.3 4.9 5.1 93.5

102 P10/P5 1.7 91 0.9 3.4 4.4 5.2 5.4 98.5

103 P5/P11 1.3 90 0.9 3.8 5.0 5.6 6.1 109.4

104 P11/P5 1.0 91 0.8 4.0 5.1 5.6 6.2 110.9

105 P5/P12 1.8 91 0.5 4.8 5.8 6.5 7.5 129.9

106 P12/P5 0.5 90 0.5 4.5 5.9 6.7 7.0 129.2

107 P5/P13 0.6 90 0.7 4.1 5.1 5.9 6.7 115.4

108 P13/P5 1.1 90 0.8 4.3 5.3 5.8 6.6 115.6

109 P5/P14 1.7 92 0.7 4.2 5.1 6.1 6.3 114.7

110 P14/P5 0.8 92 0.7 4.0 5.2 5.5 6.2 110.8

111 P6/P7 2.6 92 1.0 3.1 4.3 4.5 4.7 89.0

112 P7/P6 2.3 92 1.0 3.2 4.1 4.2 4.6 85.3

113 P6/P8 2.7 92 0.9 3.4 4.0 4.2 4.2 83.8

114 P8/P6 3.4 90 1.1 3.1 3.7 3.9 4.1 78.4

115 P6/P9 2.6 92 1.0 3.2 4.3 4.9 4.9 91.8

116 P9/P6 2.9 92 1.0 3.2 4.0 4.6 5.0 89.9

117 P6/P10 2.3 92 1.1 3.0 4.0 4.2 4.6 84.0

118 P10/P6 2.4 92 1.0 3.1 4.1 4.6 4.8 88.8

119 P6/P11 1.8 90 1.0 3.5 4.9 5.2 5.5 102.4

120 P11/P6 1.8 91 0.9 3.5 4.7 5.2 5.6 102.3

121 P6/P12 1.1 91 0.8 4.2 4.9 5.8 6.2 111.0

122 P12/P6 0.8 91 0.6 4.4 5.2 5.7 6.2 113.5

123 P6/P13 1.6 92 0.9 3.7 4.7 5.5 5.8 104.8

124 P13/P6 1.3 91 1.0 3.7 5.0 5.6 6 108.0

125 P6/P14 1.4 92 1.0 3.9 4.9 5.2 5.5 103.4

126 P14/P6 1.9 92 1.0 3.5 4.5 4.9 5.1 95.5

127 P7/P8 0.8 92 0.7 3.4 4.4 4.5 4.9 91.0

128 P8/P7 1.4 92 0.9 3.4 4.4 4.5 4.8 90.9

129 P7/P9 1.6 92 0.9 3.3 4.0 4.8 5.0 90.8

130 P9/P7 1.3 92 1.0 3.7 4.4 5.1 5.6 99.2

131 P7/P10 1.1 92 0.8 3.5 4.4 5.0 5.3 96.6

132 P10/P7 1.0 93 0.8 4.0 4.7 5.4 5.8 104.8

133 P7/P11 2.4 91 1.0 3.4 4.4 5.0 5.3 96.2

134 P11/P7 2.6 91 1.2 3.3 4.3 4.8 5.1 92.8

135 P7/P12 1.4 90 0.9 3.7 4.7 5.7 5.8 106.7

136 P12/P7 1.6 91 0.9 3.6 4.5 5.5 5.5 101.3

137 P7/P13 2.3 91 1.0 3.3 4.1 4.5 4.9 89.7

138 P13/P7 2.3 91 1.0 3.4 4.3 4.6 4.9 91.3

139 P7/P14 2.3 92 1.2 3.4 4.3 4.8 4.8 92.2

140 P14/P7 1.4 91 1.0 3.4 4.1 4.6 5.0 91.0

141 P8/P9 2.7 91 1.0 3.4 4.0 4.3 4.8 86.3

142 P9/P8 2.5 92 1.1 3.5 4.1 4.5 4.7 88.8

143 P8/P10 1.2 92 0.8 3.4 4.0 4.4 4.8 87.4

144 P10/P8 1.4 91 0.9 3.4 4.3 4.5 4.8 89.9

145 P8/P11 2.5 90 1.1 3.2 4.3 4.5 5.0 90.2

146 P11/P8 2.8 90 1.9 3.0 4.0 4.5 4.8 87.3

147 P8/P12 1.7 91 0.9 3.5 4.4 4.6 4.8 92.7

148 P12/P8 1.8 90 1.0 3.4 4.4 4.9 5.0 94.3

149 P8/P13 2.2 91 1.1 3.0 4.0 4.2 4.3 82.6

150 P13/P8 2.4 91 1.1 3.4 4.1 4.3 4.8 87.2

151 P8/P14 1.5 92 1.1 3.4 4.3 4.5 4.7 89.4

152 P14/P8 1.8 92 1.0 3.3 4.1 4.5 4.7 88.1

153 P9/P10 1.9 92 1.1 3.4 4.3 5.0 5.2 95.1

154 P10/P9 1.8 92 1.0 3.3 4.3 5.1 5.4 95.5

155 P9/P11 3.1 90 1.1 3.1 4.1 5.1 5.1 93.5

156 P11/P9 2.9 90 1.1 3.3 4.1 5.0 5.0 93.6

157 P9/P12 1.5 90 0.9 3.7 4.6 5.2 5.5 101.2

158 P12/P9 1.4 90 0.9 3.6 4.6 5.2 5.5 100.9

159 P9/P13 2.4 91 1.1 3.5 4.4 5.0 5.0 95.6

160 P13/P9 2.1 91 1.0 3.3 4.4 4.8 4.9 92.5

161 P9/P14 2.6 92 1.0 3.3 4.3 4.8 5.0 92.1

162 P14/P9 2.6 92 1.0 3.4 4.3 5.0 5.1 94.5

163 P10/P11 2.4 90 1.1 3.0 4.0 5.0 5.3 92.4

164 P11/P10 2.0 91 1.1 3.3 4.4 5.1 5.3 96.9

165 P10/P12 1.4 91 0.8 3.6 4.5 5.2 5.6 100.2

166 P12/P10 1.1 91 0.7 3.7 4.6 5.2 5.7 101.7

167 P10/P13 1.9 92 1.0 3.4 4.6 5.1 5.1 97.7

168 P13/P10 2.3 92 1.0 3.2 4.1 4.6 5.3 91.3

169 P10/P14 1.9 92 0.9 3.5 4.3 4.5 5.0 91.6

170 P14/P10 1.7 92 1.6 3.6 4.6 5.1 5.1 98.2

171 P11/P12 1.2 91 0.7 3.8 5.0 5.6 6.1 108.3

172 P12/P11 1.8 90 0.8 4.1 5.2 5.7 6.1 112.1

173 P11/P13 1.9 90 1.0 3.5 5.0 5.6 6.0 107.7

174 P13/P11 1.8 91 1.0 3.8 5.0 5.6 6.1 108.5

175 P11/P14 1.8 91 1.0 3.4 4.6 5.5 5.8 102.8

176 P14/P11 1.7 91 1.0 3.7 4.9 5.4 5.8 104.9

177 P12/P13 0.8 91 0.5 4.5 5.6 6.3 6.7 122.5

178 P13/P12 0.8 91 0.5 4.3 5.6 6.1 6.6 120.1

179 P12/P14 0.5 92 0.5 4.6 5.8 6.4 6.7 125.7

180 P14/P12 1.5 92 0.6 4.5 5.7 6.2 6.7 122.5

181 P13/P14 1.6 93 0.9 3.7 5.0 5.5 5.6 106.1

182 P14/P13 1.4 92 0.9 4.0 5.1 5.6 5.7 108.5

183 Check 1 1.6 95 0.8 4.0 5.2 5.8 6.3 113.1

184 Check 2 2.8 86 1.0 2.8 3.8 4.7 5.1 86.9

185 Check 3 3.4 84 1.2 2.7 3.7 4.4 4.8 82.9

186 Check 4 0.9 92 0.5 4.2 5.6 6.4 6.8 122.6

Mean 1.97 91.16 0.95 3.49 4.4 5.01 5.39 97.0

LSD0.05 1.2 3.09 0.45 0.5 0.5 0.58 0.62 9.95

CV 64.27 5.11 56.38 14.75 11.65 11.88 11.88 6.93

AUDPC, Area under disease progress curve; DTA, Days to anthesis; EPP, Ears per plant; GY, Grain yield; MLN1, MLN2, MLN3 and MLN4, Maize lethal necrosis disease rating at 21, 28, 35 and 42 days after inoculation, respectively.

**SUPPLEMENTAL TABLE S2** Best linear unbiased predictions (BLUP) for agronomic traits of 186 maize hybrids evaluated under rainfed conditions at Kakamega over 3 years (2020-2022).

| Hybrid | Parents | DTA (days) | DTS (days) | TLB (1‒9) | EPP | GY  (t ha^-1^) | Hybrid | Parents | DTA (days) | DTS (days) | TLB (1‒9) | EPP | GY  (t ha^-1^) |
| --- | --- | --- | --- | --- | --- | --- | --- | --- | --- | --- | --- | --- | --- |
| 1 | P1/P2 | 81 | 81 | 3.6 | 1.1 | 4.3 | 55 | P3/P6 | 78 | 82 | 4.0 | 1.3 | 3.7 |
| 2 | P2/P1 | 81 | 80 | 3.8 | 1.0 | 3.6 | 56 | P6/P3 | 79 | 80 | 4.5 | 1.0 | 3.5 |
| 3 | P1/P3 | 84 | 78 | 4.2 | 1.0 | 3.2 | 57 | P3/P7 | 80 | 79 | 3.5 | 1.0 | 4.1 |
| 4 | P3/P1 | 83 | 81 | 4.1 | 0.8 | 3.5 | 58 | P7/P3 | 80 | 80 | 3.4 | 1.0 | 4.4 |
| 5 | P1/P4 | 82 | 82 | 4.3 | 1.1 | 3.7 | 59 | P3/P8 | 81 | 84 | 4.2 | 1.1 | 3.7 |
| 6 | P4/P1 | 82 | 77 | 4.3 | 1.0 | 3.6 | 60 | P8/P3 | 82 | 84 | 4.3 | 1.0 | 4.0 |
| 7 | P1/P5 | 79 | 78 | 4.0 | 0.9 | 3.8 | 61 | P3/P9 | 78 | 83 | 3.3 | 0.8 | 4.1 |
| 8 | P5/P1 | 80 | 79 | 4.0 | 1.0 | 3.5 | 62 | P9/P3 | 78 | 78 | 3.5 | 1.0 | 3.8 |
| 9 | P1/P6 | 81 | 79 | 5.4 | 0.9 | 3.2 | 63 | P3/P10 | 81 | 77 | 4.0 | 1.1 | 3.5 |
| 10 | P6/P1 | 81 | 78 | 5.3 | 1.0 | 3.5 | 64 | P10/P3 | 81 | 75 | 3.4 | 1.1 | 4.6 |
| 11 | P1/P7 | 82 | 78 | 4.3 | 1.2 | 3.9 | 65 | P3/P11 | 78 | 77 | 3.2 | 1.0 | 4.0 |
| 12 | P7/P1 | 81 | 82 | 4.1 | 1.0 | 4.3 | 66 | P11/P3 | 77 | 78 | 2.9 | 1.0 | 4.3 |
| 13 | P1/P8 | 83 | 82 | 5.1 | 1.2 | 3.6 | 67 | P3/P12 | 76 | 77 | 2.4 | 0.9 | 4.1 |
| 14 | P8/P1 | 82 | 82 | 4.8 | 1.1 | 3.9 | 68 | P12/P3 | 77 | 80 | 2.6 | 0.9 | 3.8 |
| 15 | P1/P9 | 79 | 79 | 4.1 | 0.9 | 3.8 | 69 | P3/P13 | 78 | 78 | 2.8 | 1.2 | 4.7 |
| 16 | P9/P1 | 80 | 79 | 4.3 | 1.0 | 3.8 | 70 | P13/P3 | 79 | 80 | 4.0 | 1.2 | 4.3 |
| 17 | P1/P10 | 83 | 79 | 3.6 | 1.3 | 3.8 | 71 | P3/P14 | 83 | 81 | 3.3 | 0.8 | 3.3 |
| 18 | P10/P1 | 81 | 79 | 4.5 | 1.1 | 4.6 | 72 | P14/P3 | 81 | 77 | 3.7 | 0.9 | 3.3 |
| 19 | P1/P11 | 78 | 79 | 4.9 | 0.9 | 4.1 | 73 | P4/P5 | 80 | 78 | 4.1 | 1.1 | 3.6 |
| 20 | P11/P1 | 80 | 77 | 4.6 | 1.1 | 4.0 | 74 | P5/P4 | 78 | 81 | 3.9 | 0.9 | 4.4 |
| 21 | P1/P12 | 78 | 81 | 3.3 | 0.8 | 4.5 | 75 | P4/P6 | 79 | 79 | 5.6 | 1.0 | 3.8 |
| 22 | P12/P1 | 79 | 79 | 3.8 | 1.0 | 4.7 | 76 | P6/P4 | 79 | 79 | 5.1 | 0.9 | 4.5 |
| 23 | P1/P13 | 78 | 78 | 3.9 | 1.2 | 5.0 | 77 | P4/P7 | 80 | 81 | 4.5 | 1.2 | 4.0 |
| 24 | P13/P1 | 79 | 81 | 3.8 | 1.3 | 4.9 | 78 | P7/P4 | 81 | 83 | 4.1 | 1.0 | 4.7 |
| 25 | P1/P14 | 82 | 78 | 4.2 | 1.1 | 4.2 | 79 | P4/P8 | 81 | 83 | 5.5 | 1.0 | 4.1 |
| 26 | P14/P1 | 81 | 79 | 4.1 | 1.2 | 3.5 | 80 | P8/P4 | 81 | 84 | 4.4 | 1.2 | 4.2 |
| 27 | P2/P3 | 80 | 80 | 3.3 | 1.4 | 3.6 | 81 | P4/P9 | 79 | 76 | 4.5 | 1.1 | 4.4 |
| 28 | P3/P2 | 81 | 81 | 2.9 | 0.9 | 3.1 | 82 | P9/P4 | 78 | 76 | 4.3 | 1.3 | 3.8 |
| 29 | P2/P4 | 81 | 81 | 3.9 | 1.1 | 4.0 | 83 | P4/P10 | 81 | 77 | 4.8 | 1.1 | 4.3 |
| 30 | P4/P2 | 80 | 82 | 3.7 | 1.1 | 3.8 | 84 | P10/P4 | 80 | 76 | 4.8 | 1.0 | 3.8 |
| 31 | P2/P5 | 77 | 81 | 3.0 | 1.0 | 4.5 | 85 | P4/P11 | 78 | 79 | 4.7 | 1.2 | 4.4 |
| 32 | P5/P2 | 80 | 84 | 3.0 | 1.0 | 3.7 | 86 | P11/P4 | 79 | 79 | 4.0 | 1.0 | 4.4 |
| 33 | P2/P6 | 78 | 84 | 4.3 | 1.0 | 4.3 | 87 | P4/P12 | 79 | 77 | 4.1 | 1.1 | 4.8 |
| 34 | P6/P2 | 77 | 79 | 4.1 | 1.2 | 4.5 | 88 | P12/P4 | 78 | 78 | 3.6 | 1.1 | 4.3 |
| 35 | P2/P7 | 79 | 84 | 3.5 | 1.3 | 4.7 | 89 | P4/P13 | 79 | 79 | 3.6 | 1.1 | 4.2 |
| 36 | P7/P2 | 80 | 81 | 3.2 | 1.1 | 4.4 | 90 | P13/P4 | 78 | 82 | 3.3 | 1.0 | 4.3 |
| 37 | P2/P8 | 80 | 84 | 4.9 | 1.1 | 4.1 | 91 | P4/P14 | 82 | 80 | 3.7 | 1.0 | 4.0 |
| 38 | P8/P2 | 80 | 84 | 4.0 | 1.1 | 4.9 | 92 | P14/P4 | 83 | 81 | 3.7 | 1.1 | 4.3 |
| 39 | P2/P9 | 78 | 76 | 3.5 | 1.1 | 4.3 | 93 | P5/P6 | 80 | 81 | 4.8 | 0.9 | 3.3 |
| 40 | P9/P2 | 79 | 77 | 3.7 | 1.1 | 5.2 | 94 | P6/P5 | 81 | 80 | 5.0 | 1.2 | 4.7 |
| 41 | P2/P10 | 81 | 79 | 3.3 | 1.2 | 4.6 | 95 | P5/P7 | 80 | 81 | 3.4 | 1.0 | 4.7 |
| 42 | P10/P2 | 80 | 78 | 3.7 | 1.2 | 4.9 | 96 | P7/P5 | 80 | 80 | 3.6 | 1.2 | 4.7 |
| 43 | P2/P11 | 77 | 79 | 3.8 | 1.1 | 4.4 | 97 | P5/P8 | 81 | 80 | 4.7 | 1.0 | 4.1 |
| 44 | P11/P2 | 76 | 79 | 4.0 | 1.0 | 4.5 | 98 | P8/P5 | 81 | 80 | 4.2 | 1.0 | 4.6 |
| 45 | P2/P12 | 75 | 81 | 3.2 | 1.1 | 4.7 | 99 | P5/P9 | 78 | 79 | 4.1 | 0.8 | 4.4 |
| 46 | P12/P2 | 76 | 82 | 2.9 | 1.0 | 6.0 | 100 | P9/P5 | 79 | 80 | 4.1 | 1.0 | 5.2 |
| 47 | P2/P13 | 79 | 81 | 3.1 | 1.1 | 4.6 | 101 | P5/P10 | 81 | 78 | 3.7 | 1.0 | 4.4 |
| 48 | P13/P2 | 78 | 78 | 3.1 | 1.3 | 4.3 | 102 | P10/P5 | 81 | 81 | 4.0 | 1.2 | 4.9 |
| 49 | P2/P14 | 81 | 80 | 3.9 | 0.9 | 3.9 | 103 | P5/P11 | 78 | 82 | 4.0 | 1.1 | 4.0 |
| 50 | P14/P2 | 80 | 85 | 3.2 | 0.8 | 3.9 | 104 | P11/P5 | 79 | 83 | 3.8 | 1.0 | 4.5 |
| 51 | P3/P4 | 81 | 85 | 3.4 | 1.2 | 3.7 | 105 | P5/P12 | 79 | 81 | 2.9 | 0.9 | 4.4 |
| 52 | P4/P3 | 81 | 77 | 4.1 | 0.9 | 3.4 | 106 | P12/P5 | 78 | 83 | 3.2 | 1.0 | 4.7 |
| 53 | P3/P5 | 77 | 78 | 2.9 | 1.1 | 3.7 | 107 | P5/P13 | 78 | 82 | 3.5 | 1.1 | 4.5 |
| 54 | P5/P3 | 78 | 79 | 2.8 | 1.3 | 3.9 | 108 | P13/P5 | 77 | 79 | 3.5 | 1.1 | 4.6 |
|  |  |  |  |  |  |  |  |  |  |  |  |  |  |
| 109 | P5/P14 | 82 | 81 | 3.6 | 1.0 | 4.0 | 163 | P10/P11 | 78 | 79 | 4.6 | 1.0 | 4.3 |
| 110 | P14/P5 | 83 | 78 | 3.5 | 1.0 | 3.9 | 164 | P11/P10 | 79 | 77 | 5.0 | 1.1 | 4.1 |
| 111 | P6/P7 | 81 | 78 | 5.3 | 1.2 | 4.3 | 165 | P10/P12 | 80 | 82 | 2.9 | 1.1 | 4.2 |
| 112 | P7/P6 | 80 | 79 | 4.7 | 0.9 | 3.9 | 166 | P12/P10 | 79 | 81 | 3.7 | 1.2 | 5.1 |
| 113 | P6/P8 | 81 | 80 | 5.0 | 1.0 | 3.5 | 167 | P10/P13 | 80 | 78 | 4.5 | 1.2 | 4.5 |
| 114 | P8/P6 | 80 | 80 | 5.2 | 1.2 | 3.7 | 168 | P13/P10 | 81 | 78 | 4.1 | 1.1 | 5.1 |
| 115 | P6/P9 | 79 | 81 | 5.9 | 1.0 | 3.9 | 169 | P10/P14 | 82 | 80 | 4.4 | 1.0 | 3.9 |
| 116 | P9/P6 | 79 | 79 | 5.8 | 1.0 | 4.2 | 170 | P14/P10 | 84 | 79 | 4.4 | 1.1 | 4.2 |
| 117 | P6/P10 | 81 | 83 | 5.6 | 1.5 | 4.1 | 171 | P11/P12 | 76 | 79 | 4.0 | 1.0 | 4.1 |
| 118 | P10/P6 | 80 | 79 | 5.7 | 1.0 | 3.7 | 172 | P12/P11 | 76 | 82 | 3.4 | 1.0 | 4.6 |
| 119 | P6/P11 | 78 | 80 | 5.3 | 0.9 | 3.7 | 173 | P11/P13 | 79 | 79 | 3.8 | 1.0 | 4.5 |
| 120 | P11/P6 | 78 | 81 | 5.1 | 1.0 | 3.5 | 174 | P13/P11 | 78 | 84 | 4.2 | 1.1 | 4.7 |
| 121 | P6/P12 | 78 | 77 | 4.5 | 0.9 | 3.8 | 175 | P11/P14 | 80 | 83 | 4.5 | 1.0 | 4.6 |
| 122 | P12/P6 | 79 | 77 | 4.9 | 1.1 | 4.2 | 176 | P14/P11 | 80 | 81 | 4.0 | 1.0 | 4.4 |
| 123 | P6/P13 | 80 | 76 | 5.0 | 1.0 | 3.8 | 177 | P12/P13 | 76 | 82 | 3.6 | 0.9 | 4.7 |
| 124 | P13/P6 | 80 | 77 | 5.4 | 1.1 | 4.7 | 178 | P13/P12 | 77 | 79 | 3.7 | 1.0 | 4.0 |
| 125 | P6/P14 | 82 | 79 | 4.8 | 1.0 | 3.6 | 179 | P12/P14 | 80 | 80 | 3.1 | 0.9 | 4.8 |
| 126 | P14/P6 | 81 | 78 | 5.0 | 0.9 | 3.5 | 180 | P14/P12 | 80 | 81 | 3.4 | 1.4 | 4.4 |
| 127 | P7/P8 | 84 | 81 | 5.4 | 1.1 | 3.3 | 181 | P13/P14 | 82 | 81 | 3.4 | 1.2 | 4.6 |
| 128 | P8/P7 | 84 | 84 | 5.1 | 1.6 | 3.5 | 182 | P14/P13 | 81 | 78 | 3.8 | 1.1 | 5.5 |
| 129 | P7/P9 | 81 | 80 | 4.5 | 1.1 | 3.5 | 183 | Check 1 | 80 | 81 | 2.7 | 1.1 | 5.3 |
| 130 | P9/P7 | 83 | 81 | 4.5 | 0.8 | 3.4 | 184 | Check 2 | 73 | 73 | 4.6 | 1.0 | 5.4 |
| 131 | P7/P10 | 85 | 82 | 4.8 | 1.1 | 3.5 | 185 | Check 3 | 73 | 74 | 4.6 | 1.0 | 4.3 |
| 132 | P10/P7 | 86 | 77 | 4.2 | 1.3 | 3.5 | 186 | Check 4 | 79 | 80 | 4.4 | 1.0 | 4.1 |
| 133 | P7/P11 | 77 | 79 | 4.3 | 1.0 | 4.5 |  |  |  |  |  |  |  |
| 134 | P11/P7 | 78 | 78 | 4.4 | 1.0 | 4.0 |  |  |  |  |  |  |  |
| 135 | P7/P12 | 78 | 79 | 3.5 | 1.1 | 4.7 |  |  |  |  |  |  |  |
| 136 | P12/P7 | 78 | 78 | 3.2 | 1.1 | 4.5 |  |  |  |  |  |  |  |
| 137 | P7/P13 | 79 | 79 | 3.9 | 1.1 | 4.8 |  |  |  |  |  |  |  |
| 138 | P13/P7 | 78 | 80 | 4.0 | 1.1 | 4.8 |  |  |  |  |  |  |  |
| 139 | P7/P14 | 83 | 83 | 4.1 | 1.1 | 4.1 |  |  |  |  |  |  |  |
| 140 | P14/P7 | 82 | 82 | 4.0 | 1.3 | 4.3 |  |  |  |  |  |  |  |
| 141 | P8/P9 | 80 | 77 | 5.3 | 1.2 | 3.8 |  |  |  |  |  |  |  |
| 142 | P9/P8 | 81 | 79 | 5.2 | 1.0 | 4.1 |  |  |  |  |  |  |  |
| 143 | P8/P10 | 84 | 80 | 5.4 | 1.2 | 3.4 |  |  |  |  |  |  |  |
| 144 | P10/P8 | 84 | 80 | 5.3 | 1.1 | 3.7 |  |  |  |  |  |  |  |
| 145 | P8/P11 | 78 | 77 | 5.6 | 1.0 | 5.2 |  |  |  |  |  |  |  |
| 146 | P11/P8 | 78 | 77 | 5.4 | 1.2 | 3.6 |  |  |  |  |  |  |  |
| 147 | P8/P12 | 79 | 78 | 3.8 | 1.1 | 4.2 |  |  |  |  |  |  |  |
| 148 | P12/P8 | 81 | 78 | 4.1 | 1.1 | 4.6 |  |  |  |  |  |  |  |
| 149 | P8/P13 | 80 | 77 | 4.9 | 1.2 | 4.0 |  |  |  |  |  |  |  |
| 150 | P13/P8 | 80 | 81 | 5.2 | 1.0 | 4.2 |  |  |  |  |  |  |  |
| 151 | P8/P14 | 84 | 79 | 4.6 | 1.1 | 4.1 |  |  |  |  |  |  |  |
| 152 | P14/P8 | 85 | 81 | 4.7 | 1.4 | 3.7 |  |  |  |  |  |  |  |
| 153 | P9/P10 | 83 | 81 | 4.6 | 1.4 | 3.4 |  |  |  |  |  |  |  |
| 154 | P10/P9 | 80 | 80 | 4.6 | 1.2 | 3.8 |  |  |  |  |  |  |  |
| 155 | P9/P11 | 78 | 80 | 5.1 | 1.0 | 4.0 |  |  |  |  |  |  |  |
| 156 | P11/P9 | 76 | 80 | 4.9 | 1.0 | 4.8 |  |  |  |  |  |  |  |
| 157 | P9/P12 | 77 | 79 | 4.0 | 0.9 | 4.4 |  |  |  |  |  |  |  |
| 158 | P12/P9 | 76 | 81 | 4.2 | 1.1 | 4.5 |  |  |  |  |  |  |  |
| 159 | P9/P13 | 78 | 81 | 3.9 | 1.2 | 4.7 |  |  |  |  |  |  |  |
| 160 | P13/P9 | 78 | 82 | 3.6 | 1.0 | 4.0 |  |  |  |  |  |  |  |
| 161 | P9/P14 | 81 | 81 | 4.4 | 1.1 | 4.1 |  |  |  |  |  |  |  |
| 162 | P14/P9 | 81 | 82 | 4.4 | 1.4 | 4.5 |  |  |  |  |  |  |  |
|  | Mean | 79.8 | 79.8 | 4.1 | 1.1 | 4.2 |  |  |  |  |  |  |  |
|  | LSD_0.05_ | 2.48 | 2.91 | 1.8 | 0.4 | 1.0 |  |  |  |  |  |  |  |
|  | CV | 2.14 | 2.94 | 35.9 | 19.4 | 17.7 |  |  |  |  |  |  |  |

*, **, *** Significant at the 0.05, 0.01 and 0.001 probability levels, respectively.

DTA, Days to anthesis; DTS, Days to silking; EPP, Ears per plant; GY, Grain yield; TLB, Turcicum leaf blight.

SUPPLEMENTAL TABLE 3 Summary of ANOVA, descriptive statistics, variance component and heritability estimates of MLN disease resistance parameters, agronomic traits, and TLB for 15 tropical maize inbred lines evaluated under artificial MLN inoculation at Naivasha and under rainfed conditions at Kakamega for three seasons, 2020‒2022.

Trait Genotype G × E Mean Range LSD0.05 Genotypic variance G × E variance Residual variance Heritability

Naivasha

MLN1 *** *** 3.3 2.1‒6.0 0.8 1.09 0.30 0.05 0.93

MLN2 *** *** 4.1 2.1‒7.4 0.7 1.86 0.15 0.12 0.97

MLN3 *** *** 4.7 2.1‒8.5 0.8 2.68 0.16 0.24 0.97

MLN4 *** *** 5.1 2.1‒8.6 1.0 2.74 0.37 0.27 0.96

AUDPC *** *** 90.2 43.2‒163.3 13.5 912.83 69.59 38.01 0.97

DTA ns * 97 95‒98 n/a 4.40 47.47 96.69 0.18

EPP ns ns 0.4 0.2‒0.8 n/a 0.07 0.03 0.04 0.75

Kakamega

DTA ns ** 86 83‒87 n/a 2.35 6.32 6.62 0.42

TLB *** ns 3.3 2.4‒4.8 0.82 0.54 0.02 0.30 0.87

*, **, *** Significant at the 0.05, 0.01, and 0.001 probability levels, respectively.

n/a, not applicable; ns, not significant.

AUDPC, Area under disease progress curve; DTA, days to anthesis; DTS, days to silking; EPP, ears per plant; MLN1, MLN2, MLN3 and MLN4, maize lethal necrosis disease rating at 21, 28, 35 and 42 days after inoculation, respectively; TLB, Turcicum leaf blight.

**SUPPLEMENTAL TABLE S4** Specific combining ability for MLN2 (upper diagonal) and MLN3 (lower diagonal) disease scores of maize hybrids evaluated under artificial MLN inoculation at Naivasha over 3 years.

| Parent | | 1 | 2 | 3 | 4 | 5 | 6 | 7 | 8 | 9 | 10 | 11 | 12 | 13 | 14 |
| --- | --- | --- | --- | --- | --- | --- | --- | --- | --- | --- | --- | --- | --- | --- | --- |
| 1 | CKL18912 |  | -0.98*** | -0.36*** | -0.55*** | -1.21*** | -0.74*** | 0.40*** | 0.21* | 0.50*** | 0.33*** | 0.52*** | 0.67*** | 0.61*** | 0.61*** |
| 2 | CKL181281 | -0.80*** |  | -0.39*** | -0.57*** | -0.61*** | -0.70*** | 0.75*** | 0.60*** | 0.60*** | 0.50*** | -0.81*** | 0.43*** | 0.39*** | 0.79*** |
| 3 | CKL181379 | -0.38*** | -0.52*** |  | -0.52*** | -0.99*** | -0.65*** | 0.24** | 0.34*** | 0.34*** | 0.30** | 0.68*** | 0.55*** | 0.13 | 0.34*** |
| 4 | CKL181847 | -0.54*** | -0.50*** | -0.45*** |  | -0.87*** | -0.71*** | 0.05 | 0.21* | 0.28** | 0.42*** | 0.55*** | 0.55*** | 0.32*** | 0.84*** |
| 5 | CKL182037 | -1.03*** | -0.99*** | -1.13*** | -0.73*** |  | -0.68*** | 0.33*** | 0.37*** | 0.37*** | 0.27** | 0.77*** | 0.96*** | 0.73*** | 0.56*** |
| 6 | CKL176616 | -0.54*** | -0.93*** | -0.63*** | -0.67*** | -0.97*** |  | 0.42*** | 0.27** | 0.40*** | 0.29** | 0.80*** | 0.36*** | 0.63*** | 0.33*** |
| 7 | CKL175951 | 0.31** | 0.79*** | 0.34*** | 0.11 | 0.56*** | 0.12 |  | 0.22* | -0.15 | 0.19* | -0.31*** | -0.69*** | -0.67*** | -0.78*** |
| 8 | CKL175755 | 0.30** | 0.67*** | 0.34*** | 0.21* | 0.34*** | 0.34*** | 0.06 |  | -0.05 | -0.03 | -0.28** | -0.72*** | -0.57*** | -0.56*** |
| 9 | CKL175798 | 0.25** | 0.80*** | 0.16 | 0.06 | 0.32** | 0.56*** | -0.03 | -0.06 |  | -0.07 | -0.46*** | -0.65*** | -0.44*** | -0.67*** |
| 10 | CKL176082 | 0.12 | 0.67*** | 0.22* | 0.30** | 0.44*** | 0.18 | 0.22* | 0.00 | 0.10 |  | -0.44*** | -0.75*** | -0.48*** | -0.53*** |
| 11 | CKDHL120918 | 0.33*** | -0.94*** | 0.54*** | 0.76*** | 0.70*** | 0.76*** | -0.39*** | -0.24* | -0.20* | -0.20* |  | -0.44*** | -0.10 | -0.46*** |
| 12 | CML585 | 0.80*** | 0.45*** | 0.69*** | 0.52*** | 0.97*** | 0.53*** | -0.43*** | -0.78*** | -0.80*** | -0.81*** | -0.67*** |  | -0.17 | -0.09 |
| 13 | CKL14546 | 0.63*** | 0.36*** | 0.28** | 0.18 | 0.88*** | 0.94*** | -0.84*** | -0.68*** | -0.52*** | -0.53*** | -0.07 | -0.24* |  | -0.38*** |
| 14 | CML444 | 0.54*** | 0.93*** | 0.54*** | 0.75*** | 0.63*** | 0.31** | -0.84*** | -0.49*** | -0.64*** | -0.71*** | -0.38*** | -0.24* | -0.39*** |  |

*, **, and *** indicate significance at the 0.05, 0.01, and 0.001 levels, respectively.

**SE_ij_ = 0.089 and 0.097 for MLN2 and MLN3, respectively.**

**SUPPLEMENTAL TABLE S5** Specific combining ability for MLN4 disease scores (upper diagonal) and grain yield (t ha^-1^) (lower diagonal) of maize hybrids evaluated under artificial MLN inoculation at Naivasha over 3 years.

| Parent | | 1 | 2 | 3 | 4 | 5 | 6 | 7 | 8 | 9 | 10 | 11 | 12 | 13 | 14 |
| --- | --- | --- | --- | --- | --- | --- | --- | --- | --- | --- | --- | --- | --- | --- | --- |
| 1 | CKL18912 |  | -0.82*** | -0.48*** | -0.65*** | -1.15*** | -0.52*** | 0.45*** | 0.21* | 0.36*** | 0.42*** | 0.44*** | 0.70*** | 0.57*** | 0.45*** |
| 2 | CKL181281 | 0.83*** |  | -0.53*** | -0.19 | -0.95*** | -0.87*** | 0.54*** | 0.63*** | 0.51*** | 0.44*** | -1.04*** | 0.58*** | 0.47*** | 1.23*** |
| 3 | CKL181379 | 0.19 | 0.01 |  | -0.48*** | -1.11*** | -0.67*** | 0.31** | 0.47*** | 0.28** | -0.10 | 0.67*** | 0.78*** | 0.11 | 0.75*** |
| 4 | CKL181847 | 0.00 | 0.25 | 0.38* |  | -0.78*** | -0.65*** | -0.11 | 0.24* | 0.11 | 0.23* | 0.69*** | 0.56*** | 0.20* | 0.83*** |
| 5 | CKL182037 | 1.66*** | 1.39*** | 1.70*** | 1.28*** |  | -0.71*** | 0.32** | 0.36*** | 0.17 | 0.23* | 0.75*** | 1.11*** | 1.13*** | 0.64*** |
| 6 | CKL176616 | 1.05*** | 2.04*** | 1.21*** | 0.50** | 1.21*** |  | 0.15 | 0.12 | 0.55*** | 0.18 | 0.70*** | 0.56*** | 0.95*** | 0.21* |
| 7 | CKL175951 | -0.26 | -0.97*** | -0.03 | -0.17 | -0.66*** | -0.29 |  | 0.22* | 0.28** | 0.46*** | -0.33*** | -0.65*** | -0.76*** | -0.87*** |
| 8 | CKL175755 | -0.85*** | -0.83*** | -0.58** | -0.17 | -0.54** | 0.04 | -0.47** |  | 0.12 | 0.12 | -0.17 | -0.99*** | -0.66*** | -0.65*** |
| 9 | CKL175798 | -0.43* | -0.55** | -0.22 | 0.06 | -0.98*** | -0.52** | -0.42* | 0.61*** |  | 0.19 | -0.42*** | -0.81*** | -0.67*** | -0.66*** |
| 10 | CKL176082 | -0.35 | -0.59*** | -0.06 | -0.11 | -0.56** | -0.53** | -0.38* | -0.28 | -0.08 |  | -0.30** | -0.68*** | -0.48*** | -0.72*** |
| 11 | CKDHL120918 | -0.82*** | 0.94*** | -0.63*** | -0.79*** | -1.60*** | -1.33*** | 0.80*** | 0.77*** | 0.74*** | 0.71*** |  | -0.66*** | -0.02 | -0.32** |
| 12 | CML585 | -0.24 | -0.82*** | -0.81*** | -0.45* | -0.64*** | -1.19*** | 0.89*** | 0.99*** | 0.25 | 0.66*** | 0.59** |  | -0.22* | -0.27** |
| 13 | CKL14546 | -0.38* | -0.18 | -0.41* | -0.40* | -1.48*** | -1.36*** | 1.11*** | 0.84*** | 0.46* | 0.79*** | 0.30 | 0.15 |  | -0.63*** |
| 14 | CML444 | -0.41* | -1.55*** | -0.76*** | -0.37* | -0.78*** | -0.84*** | 0.85*** | 0.48** | 1.08*** | 0.78*** | 0.33 | 0.64*** | 0.56** |  |

*, **, and *** indicate significance at the 0.05, 0.01, and 0.001 levels, respectively.

**SE_ij_ = 0.098 and 0.176 for MLN4 and grain yield, respectively.**

**SUPPLEMENTAL TABLE S6** Specific combining ability for grain yield (t ha^-1^) (upper diagonal) and *Turcicum* leaf blight (lower diagonal) of maize hybrids evaluated under rainfed conditions.

| Parent | | 1 | 2 | 3 | 4 | 5 | 6 | 7 | 8 | 9 | 10 | 11 | 12 | 13 | 14 |
| --- | --- | --- | --- | --- | --- | --- | --- | --- | --- | --- | --- | --- | --- | --- | --- |
| 1 | CKL18912 |  | -0.22 | -0.72 | -1.03** | -0.90* | -0.64 | 0.18 | 0.37 | -0.17 | 0.69 | 0.20 | 1.02** | 1.66*** | -0.43 |
| 2 | CKL181281 | 0.15 |  | -1.81*** | -0.83* | -0.41 | 0.66 | 0.00 | 0.59 | 0.79* | 1.23** | 0.45 | 1.14** | -1.22** | -0.36 |
| 3 | CKL181379 | 0.83*** | 0.53** |  | -0.30 | -0.44 | 0.81* | 1.82*** | 0.01 | 0.37 | 0.75 | 0.64 | -0.68 | 0.44 | -0.89* |
| 4 | CKL181847 | -0.02 | 0.27 | 0.32 |  | -0.50 | 0.57 | 0.81* | 0.28 | 0.16 | -0.22 | 0.43 | 0.36 | 0.02 | 0.26 |
| 5 | CKL182037 | 0.21 | -0.14 | -0.12 | 0.16 |  | -0.20 | 1.00* | 0.66 | 1.26** | 0.60 | -0.28 | -0.31 | -0.24 | -0.24 |
| 6 | CKL176616 | 0.10 | -0.33 | -0.29 | 0.21 | 0.23 |  | 0.24 | 0.05 | 0.42 | -0.05 | -0.89* | -0.42 | -0.32 | -0.23 |
| 7 | CKL175951 | -0.09 | 0.03 | -0.42* | 0.07 | -0.20 | -0.11 |  | -1.24** | -1.47*** | -1.77*** | -0.16 | -0.19 | 0.27 | 0.50 |
| 8 | CKL175755 | -0.06 | 0.22 | 0.11 | -0.19 | -0.09 | -1.04*** | 0.44* |  | -0.15 | -1.06** | 0.54 | 0.34 | -0.36 | -0.03 |
| 9 | CKL175798 | -0.43* | -0.23 | -0.30 | -0.19 | 0.12 | 0.47* | 0.16 | 0.02 |  | -1.18** | -0.16 | 0.09 | -0.11 | 0.16 |
| 10 | CKL176082 | -0.61** | -0.28 | 0.02 | 0.43* | -0.18 | 0.21 | 0.19 | 0.22 | -0.11 |  | -0.22 | 0.20 | 0.65 | 0.37 |
| 11 | CKDHL120918 | 0.30 | -0.16 | -0.69*** | -0.12 | -0.10 | -0.09 | -0.07 | 0.50* | 0.39 | 0.17 |  | -0.65 | -0.22 | 0.33 |
| 12 | CML585 | -0.23 | 0.31 | -0.31 | 0.26 | 0.03 | 0.26 | -0.14 | -0.49* | 0.35 | -0.53* | -0.04 |  | -1.01* | 0.11 |
| 13 | CKL14546 | -0.28 | -0.16 | 0.35 | -0.75*** | 0.02 | 0.41* | 0.10 | 0.42* | -0.49* | 0.16 | -0.18 | 0.50* |  | 0.45 |
| 14 | CML444 | 0.14 | -0.21 | -0.04 | -0.45* | 0.07 | -0.04 | 0.03 | -0.07 | 0.23 | 0.30 | 0.09 | 0.01 | -0.08 |  |

*, **, and *** indicate significance at the 0.05, 0.01, and 0.001 levels, respectively.

**SE_ij_ = 0.389 and 0.202 for grain yield and *Turcicum* leaf blight, respectively.**
